# Impact of E-Beam Sterilization on Bioglass Composite Shape Memory Scaffolds

**DOI:** 10.1002/mame.202500329

**Published:** 2025-11-21

**Authors:** Brandon M. Nitschke, MaryGrace N. Wahby, Melissa A. Grunlan

**Affiliations:** 1 Department of Biomedical Engineering, Texas A&M University, College Station, Texas, USA; 2 Department of Materials Science and Engineering, Texas A&M University, College Station, Texas, USA; 3 Department of Chemistry, Texas A&M University, College Station, Texas, USA

**Keywords:** bioglass, e-beam, poly(*ε*-caprolactone), regenerative engineering, scaffold, shape memory polymer, sterilization

## Abstract

A “self-fitting” bioactive composite scaffold based on a shape memory polymer (SMP) and 45S5 Bioglass (BG) offers a promising regenerative treatment for irregularly shaped craniomaxillofacial (CMF) bone defects. Self-fitting is afforded by scaffolds prepared as crosslinked networks from *linear*-poly(*ε*-caprolactone)-diacrylate (*linear*-PCL-DA), and as semi-interpenetrating networks (semi-IPNs) with the inclusion of thermoplastic *linear*-poly(L-lactic acid) (*linear*-PLLA) (75:25 wt.%). Composites may be formed by inclusion of bioactive BG (5 and 10 wt.%) to elicit hydroxyapatite (HAp) mineralization and to increase degradation rates. To advance their clinical translation, such scaffolds must be conducive to modern sterilization methods. Electron beam (E-beam) sterilization is an attractive alternative to conventional methods (e.g., ethylene oxide). Herein, the effect of E-beam sterilization (25 kGy) on SMP composite scaffolds and non-composite controls was systematically assessed. Sterilized scaffolds largely preserved the PCL crosslinked network (i.e., similar sol contents) as well as pore features. The compressive mechanical behavior of sterilized scaffolds was also largely maintained. E-beam sterilization caused minor changes in PCL crystallinity, but excellent shape memory behavior was retained. In vitro degradation was somewhat faster following sterilization for scaffolds containing 10 wt.% BG. All E-beam sterilized composite scaffolds retained bioactivity, exhibiting HAp mineralization with exposure to simulated body fluid (SBF, 1X).

## 1 | Introduction

The treatment of complex bone defects in the craniomaxillofacial (CMF) region is a challenge due to the often irregular geometries of the defects. While autografting is considered the “gold standard” for treatment, achieving sufficient bone-to-graft contact is difficult and can lead to premature graft resorption [1, 2]. Furthermore, autografting suffers from a lack of availability and donor site morbidity [3]. A regenerative engineering approach employing an off-the-shelf scaffold is an attractive alternative for the treatment of CMF bone defects, and potentially for the restoration of other bone tissues, including orthopedic and dental types. We have previously reported “self-fitting” shape memory polymer (SMP) scaffolds from crosslinkable *linear*-poly(*ε*-caprolactone)-diacrylate (*linear*-PCL-DA, *M*_*n*_ *~* 10 kg mol^−1^, *T*_*m*_ ~55°C) [4, 5]. These SMP scaffolds were fabricated via solvent cast particulate leaching (SCPL) with a fused salt template, yielding scaffolds of high porosity (≈70%) and interconnected macropores (*d* ≈220 μm). The covalent crosslinks act as netpoints, whereas the crystalline lamellae (*T*_*trans*_ ≈55 °C) act as the switching segments [5]. When exposed to saline (*T* ≥ *T*_*trans*_), the crystalline lamellae melt and the scaffold becomes malleable, allowing it to be press-fit into an irregularly-shaped defect, and with shape recovery (*R*_*r*_) driving expansion to the defect perimeter, resulting in a conformal fit with high bone-to-scaffold contact. Upon cooling to body temperature (*T* < *T*_*trans*_), the scaffold’s rigidity is restored as the crystalline lamellae reform, and shape fixity (*R*_*f*_) locks it into this new shape. A semi-interpenetrating network (semi-IPN) SMP scaffold design, consisting of crosslinkable *linear*-PCL-DA and non-crosslinkable *linear*-poly(L-lactic acid) (*linear-PLLA*) (75:25 wt.%) demonstrated favorable accelerated degradation rates and enhanced mechanical rigidity [6]. Bioactivity, by inducing the formation of carbonated hydroxyapatite (HAp) for enhanced osteoinductivity and osseointegration [7], is highly desirable in bone tissue generation. 45S5 Bioglass (BG) is well-known for its bioactive potency and has been approved for use in numerous clinical implants [7–10]. Thus, we have also prepared BG-based composite SMP scaffolds using an SCPL method with a fused salt-BG template, resulting in the concentration of BG on the pore walls [11]. In this way, BG could be included at just 5 and 10 wt.% to achieve bioactivity (i.e., HAp mineralization in one day, 1X simulated body fluid [SBF] as confirmed by scanning electron microscopy (SEM)-electron dispersive X-ray spectroscopy (EDS)) but retained non-brittleness.

Given the potential to alter properties, the method of sterilization of an implantable device, such as scaffolds, should be carefully selected [12–16]. Heat sterilization methods, while widely utilized, may not be used on hydrolytically labile, biodegradable polymers, including the noted SMP scaffolds [17]. We have reported that ethylene oxide (EtO) sterilization did not significantly impact the properties of *linear*-PCL and semi-IPN *linear*-PCL/linear PLLA scaffolds [18]. However, there are growing health and safety concerns about EtO sterilization, and processing times are lengthy [17, 19, 20]. Thus, ionizing radiation sterilization methods have recently gained much attention. A radiation dose of 25 kGy (per ISO 11137) is known to kill microbes via ionization and guarantees a sterility assurance level (SAL) of 10^−6^ [17, 21]. Additionally, with radiation-based sterilization methods, medical implants can be terminally sterilized in their final packaging to ease processability [22]. Gamma ray sterilization requires a radioactive source (^60^Co or ^137^Cs) and may cause a change of color and odor of the implant [22, 23]. X-ray sterilization is somewhat inefficient (i.e., low conversion rates of electrons to X-rays) and expensive [24]. Electron beam (E-beam) sterilization is advantageous due to its rapid processing times and high dosage control, and so is emerging as a preferred method of radiation sterilization [23, 25]. Many current E-beam systems can penetrate up to 90 cm of lower density (e.g., ≈0.1 g cm^−3^) materials [26] such as porous scaffolds. Moreover, E-beam sterilization is compliant with industry standards (e.g., ISO 11137 for radiation sterilization of medical devices) [27] and is categorized by the FDA as an “Established Category A” method based on its historic safety and efficacy [28].

**FIGURE 1 |:**
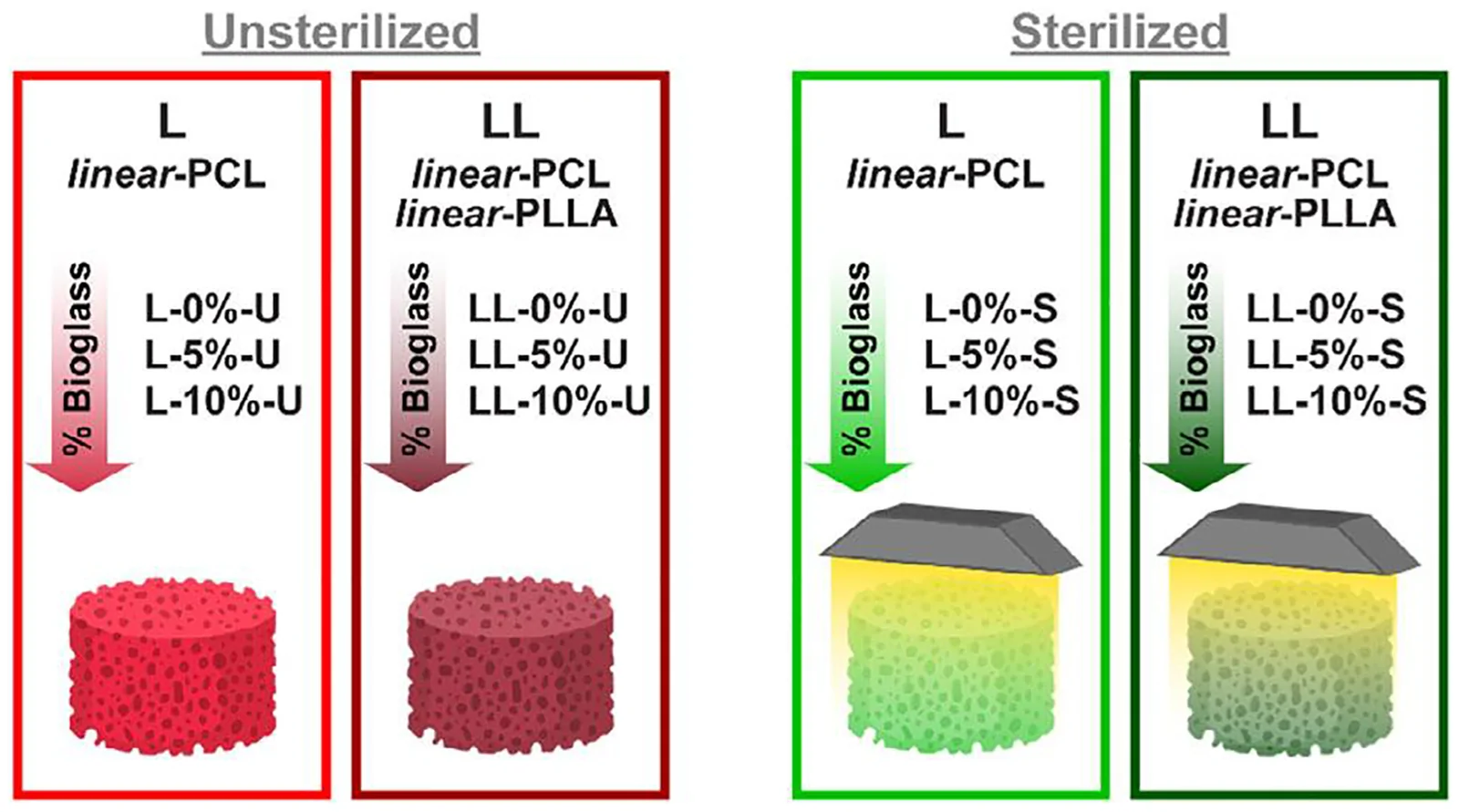
Scaffold compositions and designation: crosslinked networks prepared from linear-PCL-DA (L) and semi-IPNs prepared from linear-PCL-DA/linear-PLLA 75:25 wt.% (LL). Percentages indicate the wt.% of BG incorporated into each scaffold. “U” and “S” signify unsterilized or E-beam sterilized scaffold, respectively.

Herein, the effects of E-beam sterilization (25 kGy) on BG composite SMP scaffolds were assessed (Figure 1). A series of six scaffolds was prepared as crosslinked networks from *linear*-PCL-DA (**L**) or as semi-IPNs from *linear*-PCL-DA/*linear*-PLLA (75:25 wt.%) (**LL**), each with 5 and 10 wt.% BG, as well as without BG: **L-0%, L-5%, L-10%, LL-0%, LL-5%,** and **LL-10%.** Each composition was E-beam sterilized (**S**) and compared against the corresponding unsterilized scaffolds (**U**) in terms of crosslinking, as well as thermal, shape memory, and mechanical properties. In vitro degradation rates and bioactivity (i.e., HAp mineralization) were also evaluated.

## 2 | Materials and Methods

### 2.1 | Materials

*Linear*-PCL-diol (*linear*-PCL-diol, *M*_*n*_ = 10 kg mol^−1^ per manufacturer), 4-dimethylaminopyridine (DMAP), triethylamine (Et_3_N), acryloyl chloride, potassium carbonate (K_2_CO_3_), anhydrous magnesium sulfate (MgSO_4_), sodium chloride (NaCl), *N*-vinyl-2-pyrrolidone (NVP), 2,2-dimethoxy-2-phenylacetophenone (DMP), deuterated chloroform (CDCl_3_), sodium hydroxide (NaOH), sodium bicarbonate (NaHCO_3_), potassium chloride (KCl), potassium phosphate dibasic trihydrate (K_2_HPO_2_ · 3H_2_O), magnesium chloride hexahydrate (MgCl_2_ · 6H_2_O), hydrochloric acid (HCl), calcium chloride (CaCl_2_), sodium sulfate (Na_2_SO_4_), tris(hydroxymethyl)aminomethane (tris), and solvents were purchased from Sigma–Aldrich. Solvents were dried using 4 Å molecular sieves prior to use. Bioglass-45S5 (BG, 10 μm) was acquired from Mo-Sci Corp (Rolla, MO, USA).

### 2.2 | Syntheses

Prior to synthesis, glassware was rinsed with acetone and dried in an oven set to 120 °C. Reactions were run in a positive N_2_ environment. Successful syntheses were confirmed with ^1^H NMR spectroscopy (operating in 400 MHz in Fourier Transform mode with CDCl_3_ as the standard).

#### 2.2.1 | Linear-PCL-DA

*Linear*-PCL-diol (*M*_*n*_ = 10 kg mol^−1^) was acrylated to produce *linear*-PCL-DA per a prior report [6]. Briefly, in a round-bottom (rb) flask, 20.0 g *linear*-PCL-diol and 6.6 mg DMAP were dissolved in methylene chloride (DCM, 120 mL). After purging the flask with N_2_ for ≈3 min, Et_3_N (0.56 mL) and acryloyl chloride (0.65 mL) were successively added dropwise. The reaction was allowed to continue at room temperature (RT) for 30 min, followed by under reflux overnight (ON) at 55°C. The reaction was quenched with removal from heat, and the DCM was removed via rotary evaporation. Next, the crude product was redissolved in ethyl acetate (135 mL) and gravity filtered. Solvent was once again removed, and then the product was redissolved in DCM (140 mL) and washed with K_2_CO_3_ (13.5 mL) in a separatory funnel. After the layers were allowed to separate ON, the organic layer was collected, dried with MgSO_4_, and finally the DCM was removed. After drying under vacuum (RT, ON, 30 inches of mercury [in. Hg]), acrylation (>90%) was confirmed with ^1^H NMR and agreed with the prior report [6].

#### 2.2.2 | Linear-PLLA

*Linear*-PLLA (*M*_*n*_ = 15 kg mol^−1^) was synthesized via a ring-opening polymerization (ROP) per a prior protocol [6]. L-lactide (6.0 g), ethylene glycol (25 mg), and Sn(Oct)_2_ were combined in a rb flask, purged with N_2_ for 3 min, and allowed to react ON at 120°C. After removal from heat, the crude product was dissolved in chloroform (30 mL) and precipitated into cold methanol. The precipitate was collected via vacuum filtration and finally dried under vacuum (RT, ON, 30 in. Hg). ^1^H NMR spectra agreed with the prior report and confirmed successful synthesis [6].

### 2.3 | Scaffold Fabrication

Scaffolds were fabricated via an SCPL method employing a fused salt-BG template (a.k.a., “glass-in-salt mold”) developed in a prior report [11]. Briefly, sieved salt (10.0 g, 459 ± 70 μm) and the appropriate BG amount (0, 5, or 10 wt.%) were added into a 20 mL scintillation vial and enthusiastically mixed. DI H_2_O (0.81 mL) was added in 4 portions, with vigorous mixing after each addition. The NaCl and BG was packed down with a rod and centrifuged (3220 rpm, 15 min) to create the fused NaCl/BG template. The H_2_O was removed by drying the vials under vacuum (RT, ON, 30 in. Hg). Macromer solutions were prepared by dissolving the polymer(s) in DCM (0.16 g mL^−1^) and a photoinitiator solution (10 wt.% DMP dissolved in NVP) was added at 15 vol% and mixed on a shaker plate (150 rpm) for 3 min. Approximately 5 mL of the solution was added to each fused template and centrifuged (1260 rpm, 10 min) to facilitate diffusion throughout the template. Then, to crosslink the *linear*-PCL-DA, the vials were exposed to UV light (UV-Transilluminator, 6 mW cm^−2^, 365 nm) for 7 min under aluminum foil-covered beakers. After evaporating the DCM, NaCl was leached from the vials by soaking in water and EtOH (1:1 by vol) for 5 days, changing the solution daily. The resulting cylindrical specimens were dried under vacuum (RT, ON, 30 in. Hg), annealed under vacuum (30 in. Hg) [85 °C for ‘non-semi-IPNs’ (i.e., no *linear*-PLLA) (1 h) or at 180°C for semi-IPNs (5 min)], maintained at RT for 48 h, biopsy punched (Integra Miltex) to *d* ~ 6 mm, and then sliced into discs (*t* ~ 2 mm) (Vibratome, Leica VT 1000 S), discarding top and bottom sections (*t* ~ 2 mm). Scaffold specimen discs (*d* ~ 6 mm × *t* ~ 2 mm) were used for subsequent analyses.

### 2.4 | Film Fabrication

*Linear*-PCL-DA and semi-IPN *linear*-PCL-DA/*linear*-PLLA films were prepared for select analyses via solvent casting. To prepare BG-containing films (used only for porosity calculations), the designated BG wt.% was added to the macromer solution (prepared as previously described) and allowed to stir ON in a rb flask for dispersion. To the macromer solution (with and without BG), the photoinitiator solution was added at 15 vol% and allowed to mix with the macromer solution. A glass pipette was used to transfer the resulting solution into a silicone mold (*d* ~ 50 mm × *t* ~ 2 mm) secured with binder clips between glass slides (Corning). The mold was exposed to UV light (UV-Transilluminator, 6 mW cm^−2^, 365 nm) for 3 min per side to allow the *linear*-PCL-DA to crosslink. The cured films were removed from the mold, vacuum dried (RT, ON, 30 in. Hg), and subsequently soaked in EtOH (150 rpm, 3 h). Films were air-dried in a fume hood (RT, ON), and annealed under vacuum (30 in. Hg) at 85 °C for non-semi-IPNs (1 h) or 180 °C for semi-IPNs (5 min). Final film specimens (*d* ~23 mm × *t* ~2 mm) were then used for analyses.

### 2.5 | E-beam Sterilization

Scaffold specimens and films were placed in sealed plastic bags (clear low density polyethylene [LDPE], Reloc Zippit). An irradiation dose of 25 kGy was applied with a 10 MeV electron beam (L3 Pulse Sciences, 15 kW S band). Alanine dosimeters were placed on the top and bottom of each specimen-containing bag to confirm the dosage received. After sterilization, all scaffolds and films were stored in their packaging within a desiccator until testing.

### 2.6 | Film Characterization

#### 2.6.1 | Static Contact Angle (*θ*_static_)

The wettability of films was assessed with a static water contact angle. Briefly, a Theta Flex goniometer (Biolin Scientific) dispensed 5 μL DI H_2_O onto the surface of each film. After 1 min, the *θ*_*static*_ of each film was determined via the drop shape analysis software (OneAttension). Results reported from 3 droplets measured on a film.

#### 2.6.2 | Surface Morphology and Roughness

Film surface morphology and roughness (*arithmetical mean height, Sa*) were evaluated on unsterilized and sterilized films (without BG) at 3 different areas (*N* = 3) of a single film by a scanning confocal laser microscope (Evident LEXT OLS5100-LAF). *Sa* was calculated with OLS51-BSW software in accordance with ISO 25178 [29]. (Note: Because it does not stay suspended in the solvent-based macromer solution, BG-containing films could not be produced with uniform distribution of BG in the polymer matrices.)

### 2. 7 | Scaffold Characterization

#### 2.7.1 | Sol Content

Scaffold specimens (*N* = 3) were weighed and each submerged in 10 mL DCM within a 20 mL scintillation vial. Sealed vials were maintained for 48 h atop a shaker plate (150 rpm). Then, scaffolds were removed, briefly rinsed with DCM, and dried under vacuum (RT, ON, 30 in. Hg). Initial (*m*_*i*_) and final (*m*_*f*_) masses were used to calculate sol content per Equation 1.


(1) $$\text{sol content}(\%)=\frac{m_{i}-m_{f}}{m_{i}}\times 100$$

#### 2.7.2 | Pore Features

Scaffold specimen pore morphology and pore size were evaluated with scanning electron microscopy (SEM, Tescan Vega 3, accelerating voltage of 10 kV). Before imaging, scaffold specimen cross-sections were coated with Pt-Pd (≈10 nm). Based on SEM images (*N* = 10), the average pore size was determined from pores measured along each image diagonal midline using ImageJ. Porosity of scaffolds (*N* = 3) was determined with Equation 2, after the densities of scaffolds and films for each composition were determined gravimetrically.


(2) $$\text{Porosity}(\%)=\frac{\rho_{\text{solid film}}-\rho_{\text{porous scaffold}}}{\rho_{\text{solidfilm}}}*100$$

#### 2.7.3 | T_m_ and % Crystallinity

Differential scanning calorimetry (DSC) was used to determine the scaffold *T*_*m*_ and percent crystallinity (%*X*_*c*_) values for PCL and PLLA. Scaffold specimens (≈10 mg, *N* = 3) were sealed in aluminum hermetic pans. Non-semi-IPN scaffolds (i.e., no *linear*-PLLA) were heated from 0 °C to 100 °C (at 5 °C min^−1^), whereas semi-IPN scaffolds were heated to 200 °C (at 5 °C min^−1^) to account for the higher *T*_*m*_ of PLLA. Two cycles were performed, and the *T*_*m*_ and *%X*_*c*_ were obtained from the second cycle to eliminate thermal history. *T*_*m,PCL*,_ and *T*_*m,PLLA*_ values were determined by the maximum point of the corresponding endothermic melt peak (TA Universal Analysis software). Percent crystallinity (%*X*_*c*_) of PCL and PLLA were calculated with Equation 3:[30]

(3) $$\%X_{c}=\frac{\Delta H_{m}}{\Delta H_{c}^{\circ }*w}$$

where enthalpy of fusion (Δ*H*_*m*_) taken from the integral of the endothermic melt peak area (TA Universal Analysis software), $\Delta {H_{c}}^{\circ }$ is the melting enthalpy of theoretical 100% crystalline PCL (139.5 J g^−1^)[31] or PLLA(93.0 J g^−1^)[32], and *w* is the mass fraction of the respective polymer.

#### 2.7.4 | Shape Memory Behavior

The shape memory behavior of scaffold specimens (*N* = 3) was assessed by mimicking implantation into a circular defect. Scaffold initial diameter was measured, and the scaffold was then submerged in 60 °C water for 1 min. Next, the malleable scaffold was press-fit into an UHMWPE model defect (*d* ~ 5 mm × *t* ~ 3 mm), After cooling to RT in the mold for 3 min, the scaffold was removed and its diameter measured to assess shape fixation. Next, the scaffold was resubmerged in warm water for 1 min to allow for shape recovery, and the diameter was measured to assess shape recovery. The protocol was repeated for a second cycle to remove any thermal history of the scaffold. The following Equations 4 and 5 were used to calculate percent shape fixity (*R*_*f*_) and percent shape recovery (*R*_*r*_). Reported values from the second cycle (*N* = 2) using the following equations:

(4) $$R_{f}(N)=\frac{\varepsilon_{u}(N)}{\varepsilon_{m}}*100$$

(5) $$R_{r}(N)=\frac{\varepsilon_{i}(N)}{\varepsilon_{r}(N)}*100$$

where *ε*_u_(*N*) is the scaffold diameter after being press-fit into the mold, *ε*_*m*_ is the diameter of the mold, *ε*_*i*_(*N*) is the diameter after shape-recovering from the mold, and *ε*_*r*_(*N*) is the initial diameter of the scaffold. All diameters were measured via a caliper with a resolution of 0.01 mm. The defect diameter was not altered during the analysis.

#### 2.7.5 | Compression Testing

Scaffold specimens (*N* = 5) were subjected to static compression testing (Intron 5944). After pre-loading each scaffold with a force of ≈0.1 N, a constant strain (1.5 mm min^−1^) was applied up to 85%. Compressive modulus (*E*) was calculated from the initial linear region of the curve (0%–10% strain) using a custom MATLAB code. Compressive strength (*CS*) was determined from the stress at 85% strain. Toughness was also determined via the MATLAB code by integrating the stress–strain curve up to 85% strain.

#### 2.7.6 | Degradation

Degradation testing was performed under accelerated conditions (0.1 M NaOH, 37°C) in accordance with ASTM F1635 [33]. Scaffold specimens (*N* = 3 per each time-point noted below) were weighed, individually placed in a 20 mL scintillation vial, and 10 mL of NaOH solution was added. All vials were then placed in a benchtop shaking incubator (VWR 1570, 120 rpm). At 3, 6, 8, 10, 12, 14, and 17 day time-points, scaffold specimens were removed from the vials, gently rinsed with DI water, and dried under vacuum (RT, 48 h, 30 in. Hg). The final scaffold masses were measured, and gravimetric mass loss was calculated.

#### 2.7.7 | Bioactivity

Bioactivity testing for HAp mineralization was performed in SBF (1X), per Kokubo [34]. Each scaffold specimen (*N* = 3) was added to a 50 mL centrifuge tube along with 10 mL of SBF. Centrifuge tubes were then placed in a water bath set to 37°C for the allotted time of 1 day and 2 weeks. At each time-point, the scaffolds were removed from the centrifuge tubes, rinsed with DI water, patted dry with a Kimwipe, and dried under vacuum (RT, ON, 30 in. Hg). Each scaffold specimen was coated with 10.0 nm Pt-Pd and imaged using SEM (Tescan Vega 3, accelerating voltage of 10 kV). Energy-dispersive X-ray spectroscopy (EDS, Oxford Instruments, 10 kV) was used to analyze Ca/P molar ratios of HAp mineralization on each scaffold surface.

### 2.8 | Statistical Analyses

Data were reported as an average ± standard deviation and were analyzed in GraphPad Prism with a one-way ANOVA test. A *p*-value < 0.05 was considered statistically significant.

## 3 | Results and Discussion

### 3.1 | Crosslinking and Pore Morphology

To evaluate the potential impact of E-beam sterilization, a series of 4 BG-based composite SMP scaffolds and 2 non-composite controls were prepared (Figure 1). These were based on *linear*-PCL-DA **(L)** (to form crosslinked networks) **(L-0%, L-5%,** and **L-10%)** or from *linear*-PCL-DA*/linear*-PLLA (75:25 wt.%) (to form semi-IPNs) **(LL-0%, LL-5%,** and **LL-10%)**. BG was incorporated at 0, 5, and 10 wt.%. Composite scaffolds were fabricated via SCPL, where the fused salt-BG template resulted in BG concentrated on the pore walls [11]. Non-composite scaffolds were likewise prepared with a fused salt template (i.e., no BG). Scaffolds were subsequently E-beam sterilized (25 kGy) **(S)**, and unsterilized scaffolds **(U)** were used for comparisons.

Since E-beam sterilization has been shown to cause chain scission of uncrosslinked (i.e., thermoplastic) PCL [35, 36], sol content testing was performed to assess the potential impact to the crosslinked PCL network, including those that comprise the semi-IPNs. Overall, there was no difference between sterilized and unsterilized scaffolds of the same composition (Figure S1 and Table S1). Crosslinked network scaffolds had similarly low sol values (<10%) to unsterilized scaffolds, indicating that the PCL crosslinked network was not compromised. For semi-IPNs, unsterilized scaffolds exhibited sol content values (≈23%–28%) that corresponded to the amount of incorporated uncrosslinked *linear*-PLLA. After E-beam sterilization, sol content values remained statistically similar. Thus, E-beam sterilization did not appreciably compromise the PCL crosslinked network of the composite scaffolds, as well as of the non-composite controls. Yet, it is important to note that a lack of increase in sol content does not confirm an absence of chain scission. While this would be the case for thermoplastic PCL, in the case of crosslinked PCL, chain scission may result in chains still bound to the network and thereby not extracted as sol with solvent.

**FIGURE 2 |:**
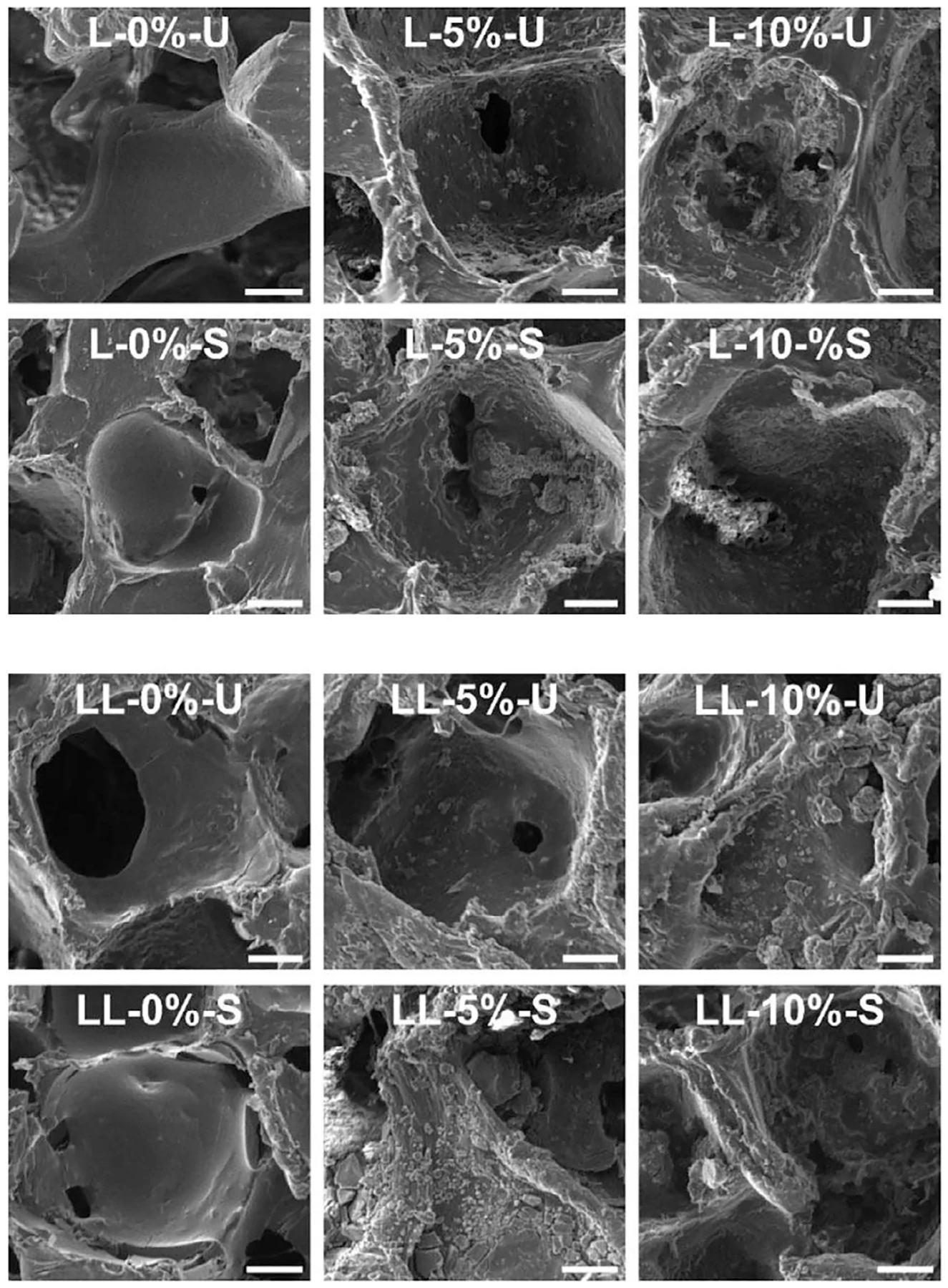
SEM images of scaffolds: Crosslinked networks prepared from linear-PCL-DA (L) and semi-IPNs prepared from linear-PCL-DA/linear-PLLA 75:25 wt.% (LL). Percentages indicate the wt.% of BG incorporated into each scaffold. “U” (unsterilized) and “S” (E-beam sterilized). (Scale bars = 50 μm).

As noted, fused salt-BG and salt templates were used to fabricate composite and non-composite scaffolds, respectively, possessing interconnected macropores, which is essential to support bone regeneration [37]. Thus, the impact of E-beam sterilization on scaffold pore features was assessed. Following E-beam sterilization, scaffolds maintained statistically similar macropore sizes (≈230–270 μm) and high porosities (60%−70%) (Figure 2; Figure S3 and Table S2). This was expected based on the aforementioned overloading maintenance of the PCL crosslinked network following E-beam sterilization.

### 3.2 | Compressive Mechanical Properties

**FIGURE 3 |:**
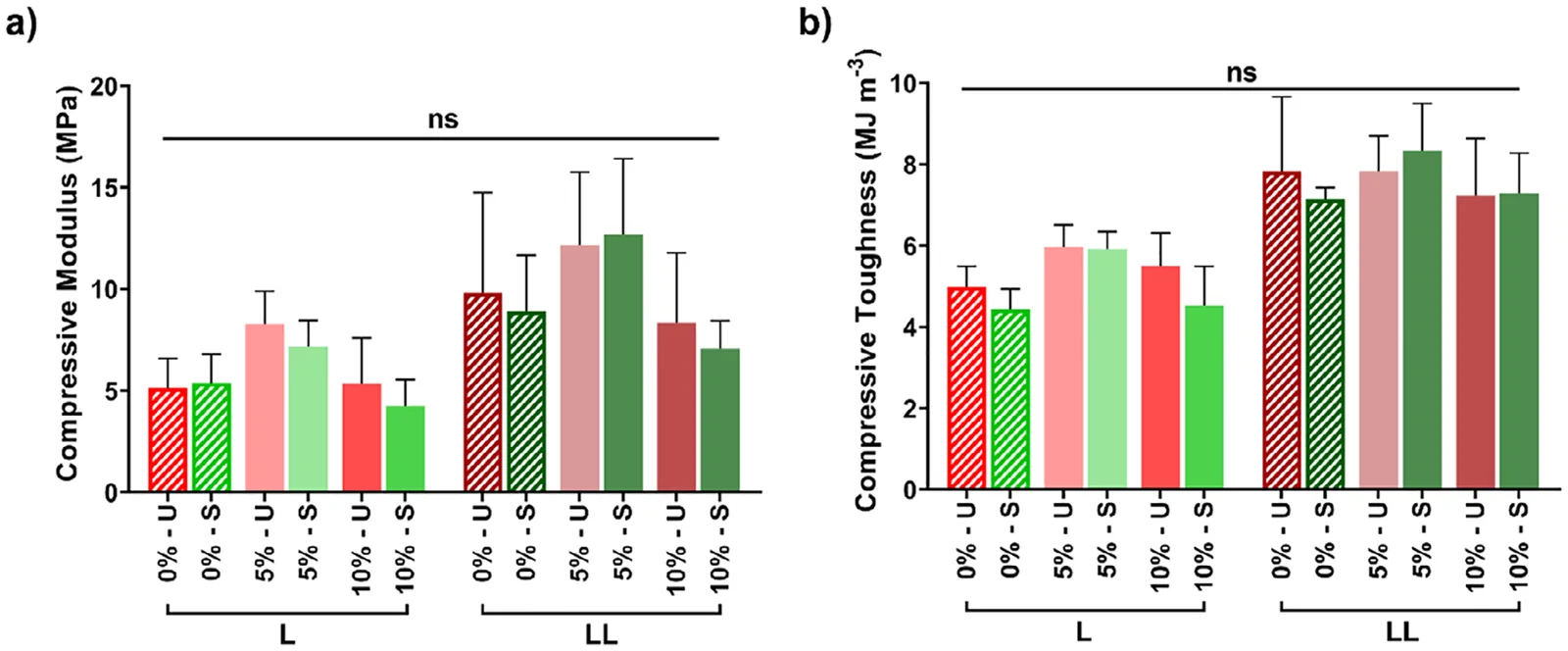
Scaffold compressive (a) E and (b) toughness: Crosslinked networks prepared from linear-PCL-DA (L) and semi-IPNs prepared from linear-PCL-DA/linear-PLLA 75:25 wt.% (LL). Percentages indicate the wt.% of BG incorporated into each scaffold. “U” (unsterilized) and “S” (E-beam sterilized). ns = no statistical difference vs. analogous unsterilized scaffold.

As previously reported [38], non-composites, semi-IPN scaffolds exhibited greater modulus (*E*) versus crosslinked network scaffolds (Figure 3a; Table S3), attributed to the high *T*_*g*_, semi-crystalline *linear*-PLLA. For composite scaffolds with 5 wt.% BG, *E* values modestly increased owing to the low BG concentration and localization on pore walls [11]. With 10 wt.% BG, *E* slightly decreased owing to a reduction in pore wall integrity. Still, all of these *E* values are within the range of trabecular bone [39]. Similar trends in compressive strength (*CS*) were also observed (Figure S4 and Table S3). Furthermore, all scaffolds were non-brittle and tough (Figure 3b; Table S3), withstanding compressive strains of 85%, and so would be expected to resist post-surgical fracture. Thus, it is critical that sterilization does not contribute to the diminished mechanical robustness of these scaffolds. Following E-beam sterilization, there were no significant changes in *E, CS*, or toughness of a given composite scaffold, as well as for non-composite scaffolds (Figure 3; Figure S4 and Table S3). This retention of mechanical properties following E-beam sterilization is attributed to a relatively undisrupted PCL crosslinked network, as well as retention of PCL and PLLA crystallinity as described below.

### 3.3 | Thermal Properties

**FIGURE 4 |:**
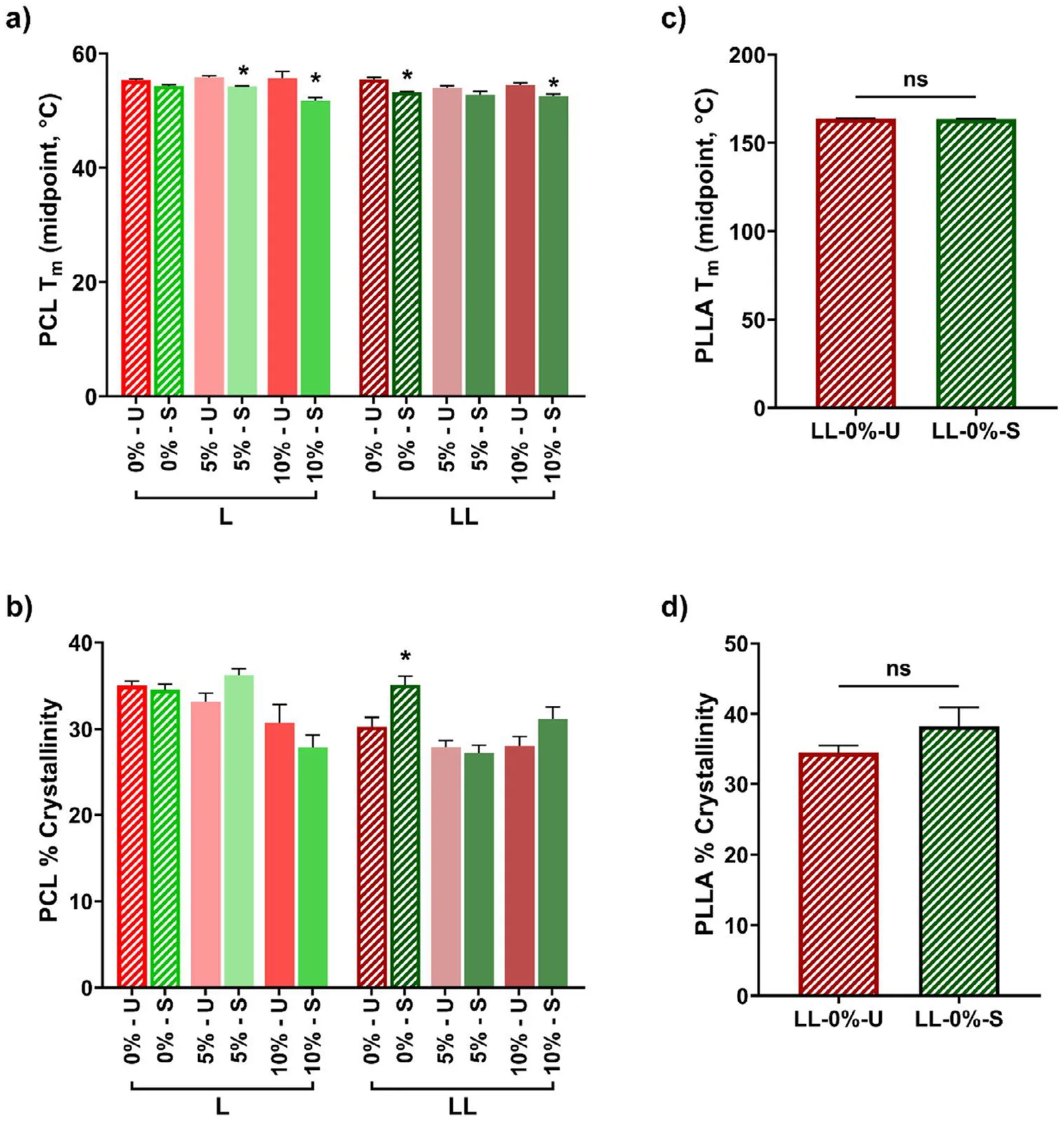
Scaffold crystallinity: Crosslinked networks prepared from linear-PCL-DA (L) and semi-IPNs prepared from linear-PCL-DA/linear-PLLA 75:25 wt.% (LL). Percentages indicate the wt.% of BG incorporated into each scaffold. “U” (unsterilized) and “S” (E-beam sterilized). (a) PCL T_m_, (b) PCL %X_c_, (c) PLLA T_m_, and (d) PLLA %X_c_; *p < 0.05 vs. analogous unsterilized scaffold; ns = no statistical difference vs. analogous unsterilized scaffold.

Radiation sterilization of thermoplastic PCL has been demonstrated to lead to chain scission, resulting in decreased % crystallinity (%*X*_*c*_) and *T*_*m*_ [40]. Conversely, other reports note that radiation-induced PCL chain scission has the opposite effect, owing to more efficient molecular folding by shorter chains [35]. Scaffold PCL crystallinity (of crosslinked networks and semi-IPNs) and PLLA crystallinity (of semi-IPNs) play crucial roles in key properties. For instance, a decrease in crystallinity could lead to reduced mechanical robustness and unanticipated accelerated degradation. For both types of scaffolds, the PCL *T*_*m*_ value defines the transition temperature (*T*_*trans*_) at which shape change is modulated. Thus, to be a viable sterilization method, E-beam sterilization should not substantially impact scaffold crystallinity. Scaffold PCL and PLLA crystallinity was therefore evaluated via DSC before and after E-beam sterilization (Figure 4; Figure S5 and Table S4). For all unsterilized scaffolds [11], the PCL *T*_*m*_ values were similar (≈55°C), but % crystallinity (%*X*_*c*_) decreased somewhat with the inclusion of 5 and 10 wt.% BG (%*X*_*c*_ ≈31%−33%, for crosslinked networks versus ≈35% for **L-0%;** *%X*_*c*_ *≈*28%, for semi-IPNs versus ≈30% for **LL-0%).** For unsterilized semi-IPN scaffolds, while PLLA crystallinity (*T*_*m*_ *~*164°C; *%X*_*c*_ *~*35%) was observed for non-composite scaffolds, it was absent in composite scaffolds. This was attributed to the physical interaction between PLLA and BG, an effect observed in other polyester composites [41–43]. After E-beam sterilization, the PCL *T*_*m*_ was statistically lower (by up to ≈3°C) for a select non-composite (**LL-0%-S**) and several composites (**L-5%-S, L-10%-S, LL-10%-S**). In terms of PCL %*X*_*c*_, only the **LL-0%** scaffold had a statistically different (≈5% increase) in PCL crystallinity after sterilization. Sterilized composite semi-IPN scaffolds retained a lack of PLLA crystallinity. For the non-composite semi-IPN scaffold (**LL-0%**), the PLLA *T*_*m*_ and %*X*_*c*_, were not altered post-sterilization. Overall, E-beam sterilization had a minimal effect on scaffold crystallinity.

### 3.4 | Shape Memory Properties

The impact of E-beam sterilization on scaffold shape fixity (*R*_*f*_) and shape recovery (*R*_*r*_) was also evaluated. As noted earlier, the PCL covalent crosslinks act as netpoints, whereas the crystalline lamellae act as the switching segments. Sol content studies verified that PCL networks were not appreciably compromised following sterilization (i.e., chain scission extensive enough to increase sol content was lacking) (Figure S1 and Table S1). For all sterilized scaffolds, while the PCL *T*_*m*_ (i.e., *T*_*trans*_) did not vary substantially (≈52–55 °C), several scaffolds exhibited slight decreases (up to ≈5%) in PCL % crystallinity (Figure 4; Figure S5 and Table S4). However, all sterilized scaffolds exhibited excellent values of *R*_*r*_ and *R*_*r*_ (>≈95%), with no reduction versus that of the corresponding unsterilized scaffold (Figure S6 and Table S5). Thus, E-beam sterilization caused no change to scaffold shape memory behavior, attributed to the sufficient retention of the PCL network and crystallinity. This is expected to afford their self-fitting behavior useful in the treatment of irregularly shaped CMF bone defects.

### 3.5 | Degradation

**FIGURE 5 |:**
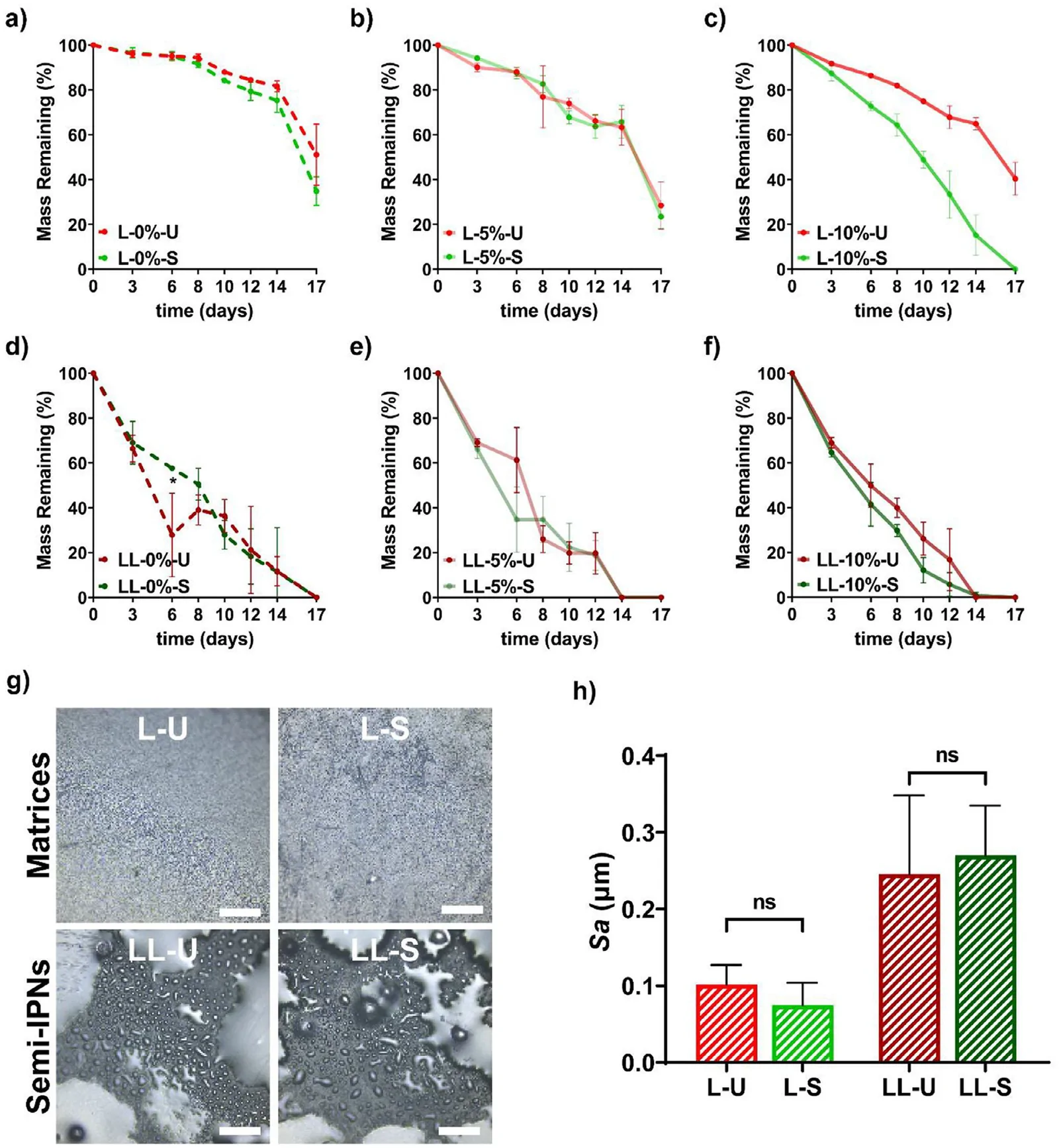
(a–f) Scaffold mass remaining (%) over time (0.1 m NaOH, 37 °C): Crosslinked networks prepared from linear-PCL-DA (L) and semi-IPNs prepared from linear-PCL-DA/linear-PLLA 75:25 wt.% (LL). Percentages indicate the wt.% of BG incorporated into each scaffold. “U” (unsterilized) and “S” (E-beam sterilized). (g) Microscopy images of analogous films (scale bars = 250 μm), and corresponding (h) surface roughness (Sa). ns = no statistical difference vs. analogous unsterilized film.

Reports on the impact of radiation on PCL has been limited to thermoplastic systems, and vary regarding the impact to degradation rates. Cottam et al. reported slower degradation rates of thermoplastic PCL films after 30.8 kGy gamma irradiation [44]. On the other hand, Bruyas et al. reported that 25 kGy E-beam irradiation increased the degradation rates of *β*-tricalcium phosphate, thermoplastic PCL composite scaffolds [36]. Herein, the degradation rates of crosslinked network and semi-IPN scaffolds, before and after E-beam sterilization, were assessed in vitro (0.1 M NaOH, 37 °C) in accordance with ASTM F1635 (Figure 5; Table S6). As previously noted, unsterilized semi-IPN scaffolds degraded faster versus crosslinked network scaffolds owing to phase separation effects (i.e., immiscibility of PCL and PLLA) that led to increased water uptake and subsequent hydrolysis [6]. Additionally, unsterilized composite scaffolds degraded faster versus the corresponding non-composite scaffold, and rates increased with greater BG content [11]. This was ascribed to the hydrophilicity of BG and the resulting water uptake into composite scaffolds [45]. For semi-IPN composite scaffolds, the lack of PLLA crystallinity also contributed to faster degradation. Following E-beam sterilization, only composite scaffolds with the highest BG content (10 wt.%) **(L-10%, LL-10%)** displayed faster degradation rates versus the corresponding unsterilized scaffold. It is hypothesized that the greater water uptake induced by higher levels of BG magnified the hydrolytic cleavage of chains that had undergone some degree of chain scission after E-beam sterilization, producing faster degradation rates. Since % crystallinity has a substantial impact on polyester degradation rates [46], the lack of dramatic changes in degradation rates post-sterilization can also be linked to coincident nominal changes to PCL % crystallinity, as well as that of PLLA % crystallinity for LL-10%. While degradation studies of biomaterials are frequently performed in vitro under such base-catalyzed conditions to reduce study duration, studies conducted at a physiological pH with both gravimetric and spectroscopic analysis (e.g., FTIR) [47] and ultimately in vivo assessment would be insightful [46].

Using BG-free analogous films, the impact of E-beam sterilization on the hydrophilicity of the polymer matrixes of the scaffolds was also assessed via contact angle analysis, but showed no change versus when unsterilized (Figure S7 and Table S7). Surface morphology and surface roughness (*Sa*) were also evaluated (Figure 5). The semi-IPN films showed the expected phase separation versus crosslinked network films, owing to the previously observed immiscibility of PCL and PLLA [6]. However, following E-beam sterilization, there were no differences between unsterilized and sterilized films.

### 3.6 | Bioactivity

**FIGURE 6 |:**
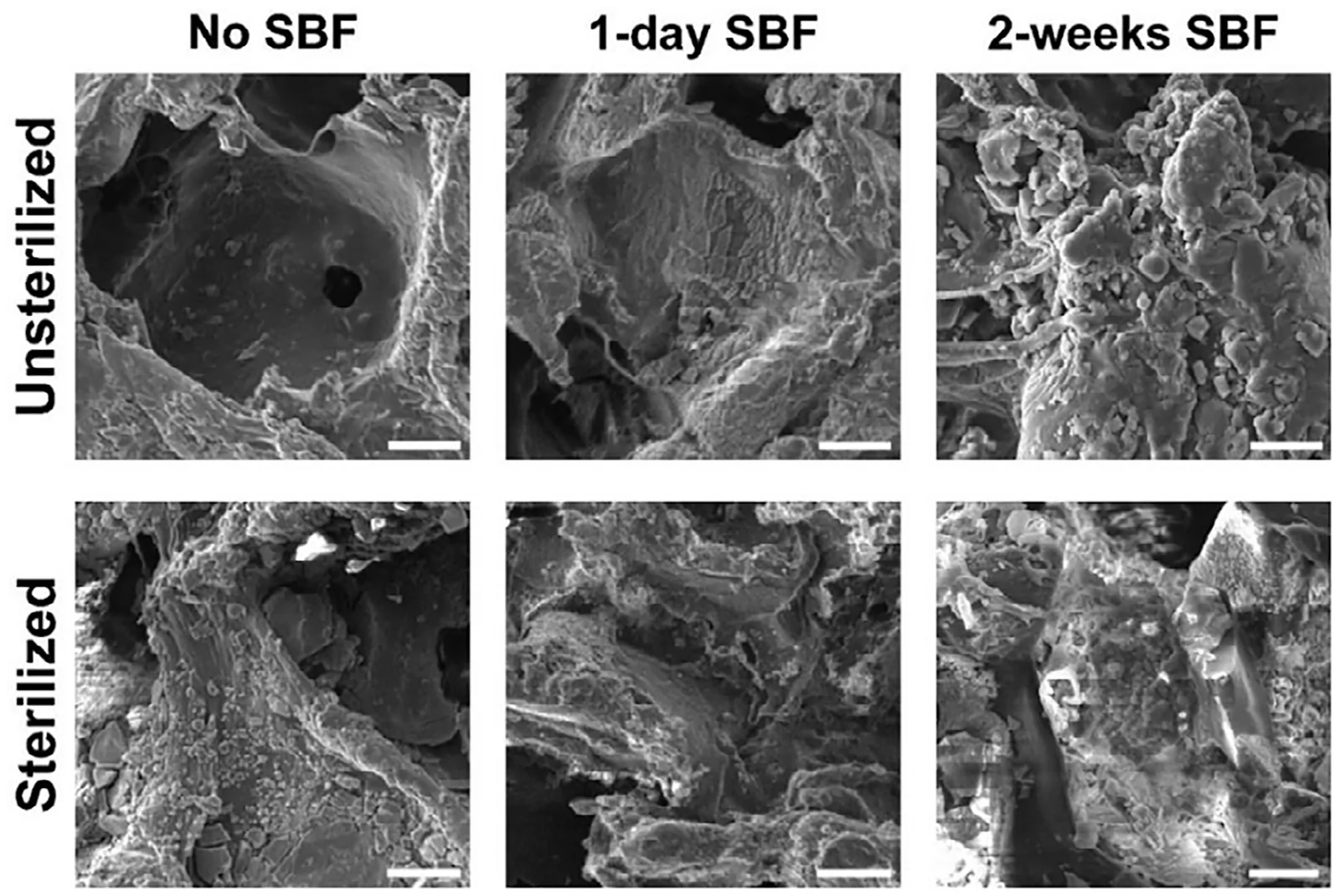
SEM images of HAp mineralization on unsterilized and sterilized LL-5% scaffolds. (Scale bars= 50 μm).

Following E-beam sterilization, it is critical that these composite SMP scaffolds retain the ability to induce HAp mineralization, known to promote ossteointegration and osteoinductivity [7]. Scaffold bioactivity was assessed by immersion in SBF (1X). SEM images of scaffolds prior to SBF exposure (Figure 2), after 1 day in SBF (Figure S8), and after 2 weeks in SBF (Figure S9) were used to visualize HAp mineralization. This temporal process is highlighted for the composite semi-IPN LL-5% in Figure 6, and EDS was used to confirm the presence of HAp (Ca/P molar ratio ≈1.67) [48] (Figure S10). Following E-beam sterilization, composite scaffolds showed no discernible differences in HAp mineralization, with non-composite scaffolds expectedly not undergoing mineralization.

## 4 | Conclusion

A regenerative engineering approach to heal irregularly shaped CMF bone defects requires a scaffold capable of conformally fitting for good tissue contact. “Self-fitting” bioactive SMP scaffolds represent a promising option. We have previously reported SMP scaffolds prepared as crosslinked networks from *linear*-PCL-DA, and as semi-IPNs from *linear*-PCL-DA/*linear*-PLLA (75:25 wt.%). While all were mechanically robust, semi-IPN scaffolds afforded a somewhat higher *E*, and a faster degradation rate. To impart bioactivity, SMP composite scaffolds were subsequently prepared by introducing BG (5 and 10 wt.%). These were fabricated using a fused salt-BG template, resulting in BG concentrated at the pore walls. Herein, the impact of E-beam sterilization (25 kGy, ISO 11137) was systematically assessed for these crosslinked network and semi-IPN composite scaffolds, as well as for non-composite scaffold controls. A lack of change in sol content indicated that the PCL crosslink networks (comprising the crosslinked networks and semi-IPNs) were not appreciably disrupted. Pore features (e.g., pore size and % porosity) were also not impacted by E-beam sterilization. After E-beam sterilization, select scaffolds exhibited slightly reduced values of PCL *T*_*m*_ (up to ≈3°C lower), and only one scaffold exhibited a change in PCL *%X*_*c*_ (≈5% increase). For the semi-IPN non-composite scaffold, PLLA crystallinity was not impacted by E-beam sterilization, and semi-IPN composite scaffolds likewise maintained a lack of PLLA crystallinity. Based on the minimal impact to the PCL network and to PLLA, sterilized scaffolds preserved their mechanical properties, and exhibited excellent shape memory behavior. In terms of in vitro degradation rates (0.1 M NaOH, 37°C), crosslinked network and semi-IPN composite SMP scaffolds with the highest BG content (10 wt.%) exhibited faster rates following E-beam sterilization. This effect is attributed to the previous observation with non-sterilized scaffolds, wherein inclusion of hydrophilic BG led to faster degradation, owing to greater water uptake to impart hydrolysis. While sol content did not increase after E-beam sterilization, some extent of chain scission may have occurred to produce ‘dangling PCL chains’ still bound to the PCL network, and such chains were more prone to hydrolysis. Finally, E-beam sterilization did not produce a discernible impact on in vitro bioactivity of composite scaffolds, and HAp occurred at just 1 day (1X SBF). Overall, these results indicate the suitability of E-beam sterilization for BG composite SMP scaffolds, as well as for analogous non-composite SMP scaffolds. Compared to our prior report on EtO-sterilized non-composite scaffolds, E-beam sterilization likewise retained pore features and shape memory behavior, and had nominal effects on degradation rates. Future studies to further probe potential E-beam associated changes to PCL network crosslink density and surface changes, and to evaluate changes to scaffold degradation and mechanical properties under physiological conditions will be informative. For clinical translation, E-beam sterilized SMP composite scaffolds should undergo biocompatibility assessment per ISO-1993.

## Supplementary Material

Supporting Information

Additional supporting information can be found online in the Supporting Information section.

**Supporting file:** mame70149-sup-0001-SuppMat.pdf

## Data Availability

Raw data is available via the Texas Data Repository (TDR).
