# Supplementary material for: Impact of E-Beam Sterilization on Bioglass Composite Shape Memory Scaffolds
Source: Macromol Mater Eng. Author manuscript; Available in PMC 2026 Sep 10. (PMC13557188; doi:10.1002/mame.202500329)
Supplement: Supplementary file 1 [file NIHMS2207313-supplement-Supporting_Information.pdf]

Supporting Information

Impact of E-Beam Sterilization on Bioglass Composite Shape Memory Scaffolds

*Brandon M. Nitschke,[1] MaryGrace N. Wahby,[1] and Melissa A. Grunlan[1,2,3]**

[1]Department of Biomedical Engineering, Texas A&M University, College Station, Texas 77843, United States.

[2]Department of Materials Science and Engineering, Texas A&M University, College Station, Texas 77843, United States.

[3]Department of Chemistry, Texas A&M University, College Station, Texas 77843, United States.

18 Pages

10 Figures

8 Tables

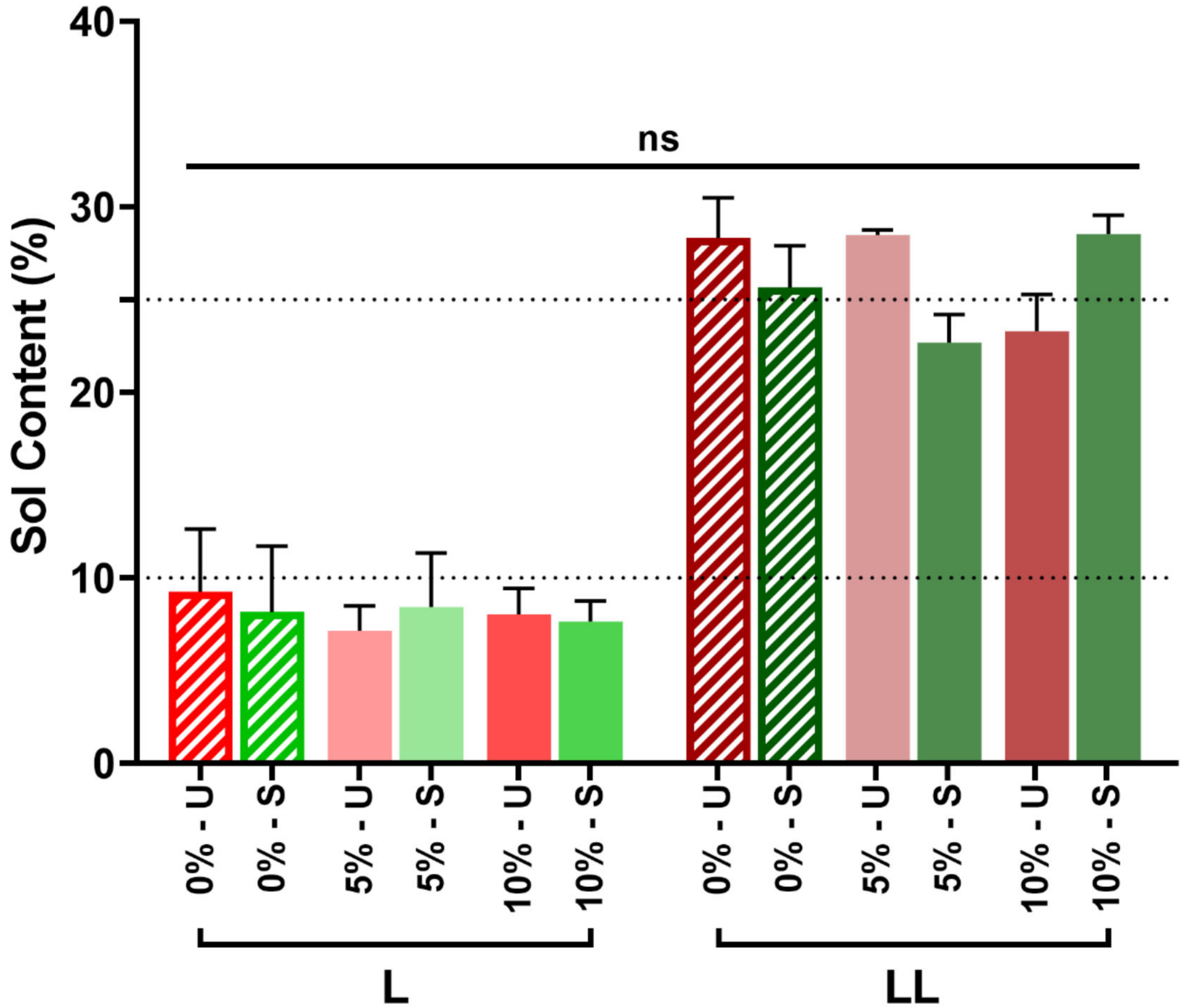

Figure S1. Scaffold sol content (%) values: Crosslinked networks prepared from *linear*-PCL-DA (**L**) and semi-IPNs prepared from *linear*-PCL-DA/*linear*-PLLA 75:25 wt% (**LL**). Percentages indicate the wt% of BG incorporated into each scaffold. "**U**" (unsterilized) and "**S**" (E-beam sterilized)*. ns* = no statistical difference vs. unsterilized composition

Table S1. Scaffold sol content (%) values: Crosslinked networks prepared from *linear*-PCL-DA (**L**) and semi-IPNs prepared from *linear*-PCL-DA/*linear*-PLLA 75:25 wt% (**LL**). Percentages indicate the wt% of BG incorporated into each scaffold. "**U**" (unsterilized) and "**S**" (E-beam sterilized).

Scaffold	Sol content (%)
**L-0%-U**	9.25 ± 3.37
**L-0%-S**	8.15 ± 3.55
**L-5%-U**	7.15 ± 1.35
**L-5%-S**	8.43 ± 2.91
**L-10%-U**	8.04 ± 1.40
**L-10%-S**	7.65 ± 1.10
**LL-0%-U**	28.32 ± 2.18
**LL-0%-S**	25.65 ± 2.25
**LL-5%-U**	28.47 ± 0.28
**LL-5%-S**	22.69 ± 1.50
**LL-10%-U**	23.29 ± 1.99
**LL-10%-S**	28.53 ± 1.01

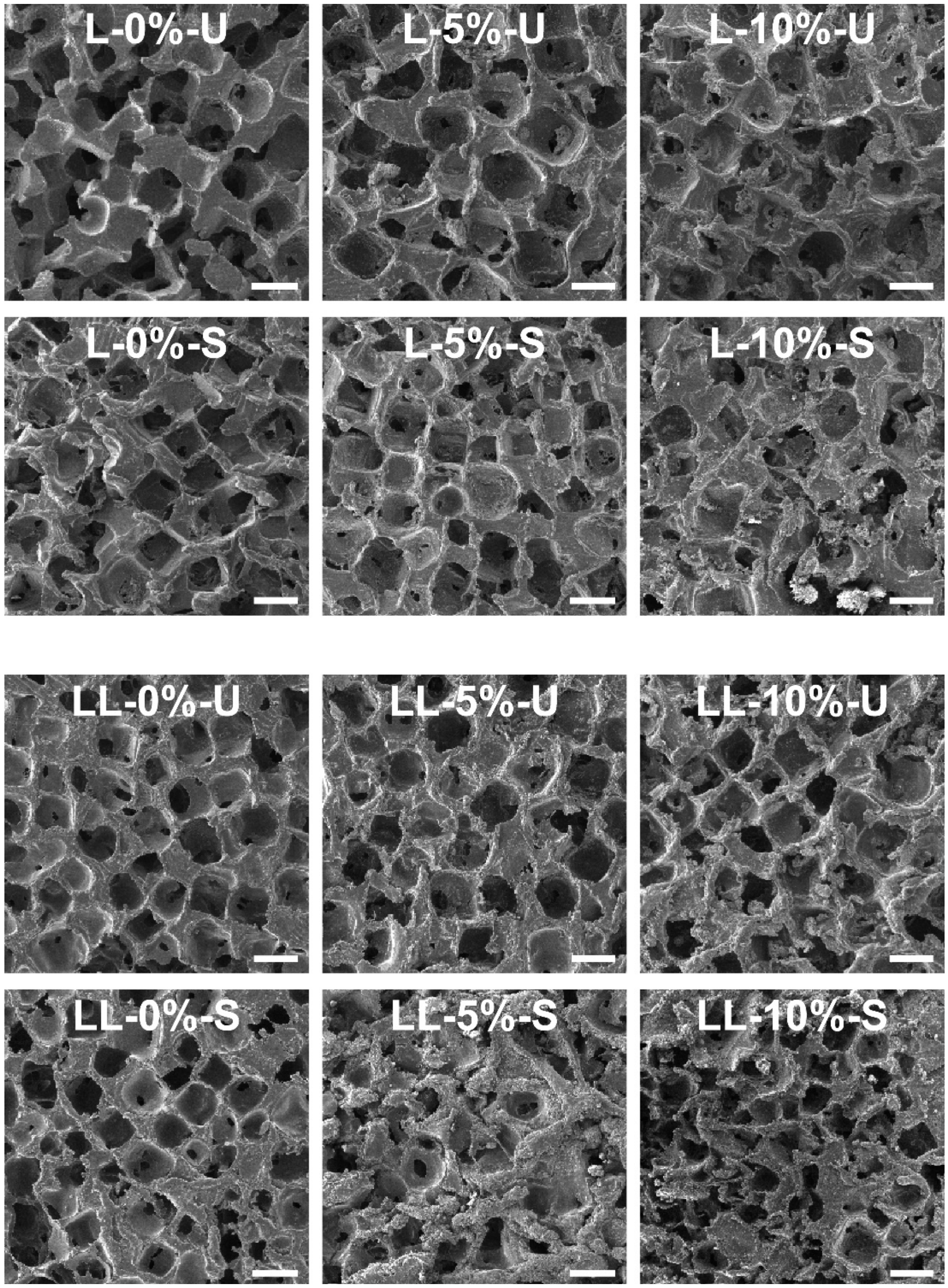

Figure S2. SEM images of scaffolds: Crosslinked networks prepared from *linear*-PCL-DA (**L**) and semi-IPNs prepared from *linear*-PCL-DA/*linear*-PLLA 75:25 wt% (**LL**). Percentages indicate the wt% of BG incorporated into each scaffold. "**U**" (unsterilized) and "**S**" (E-beam sterilized). (Scale bars = 200 μm)

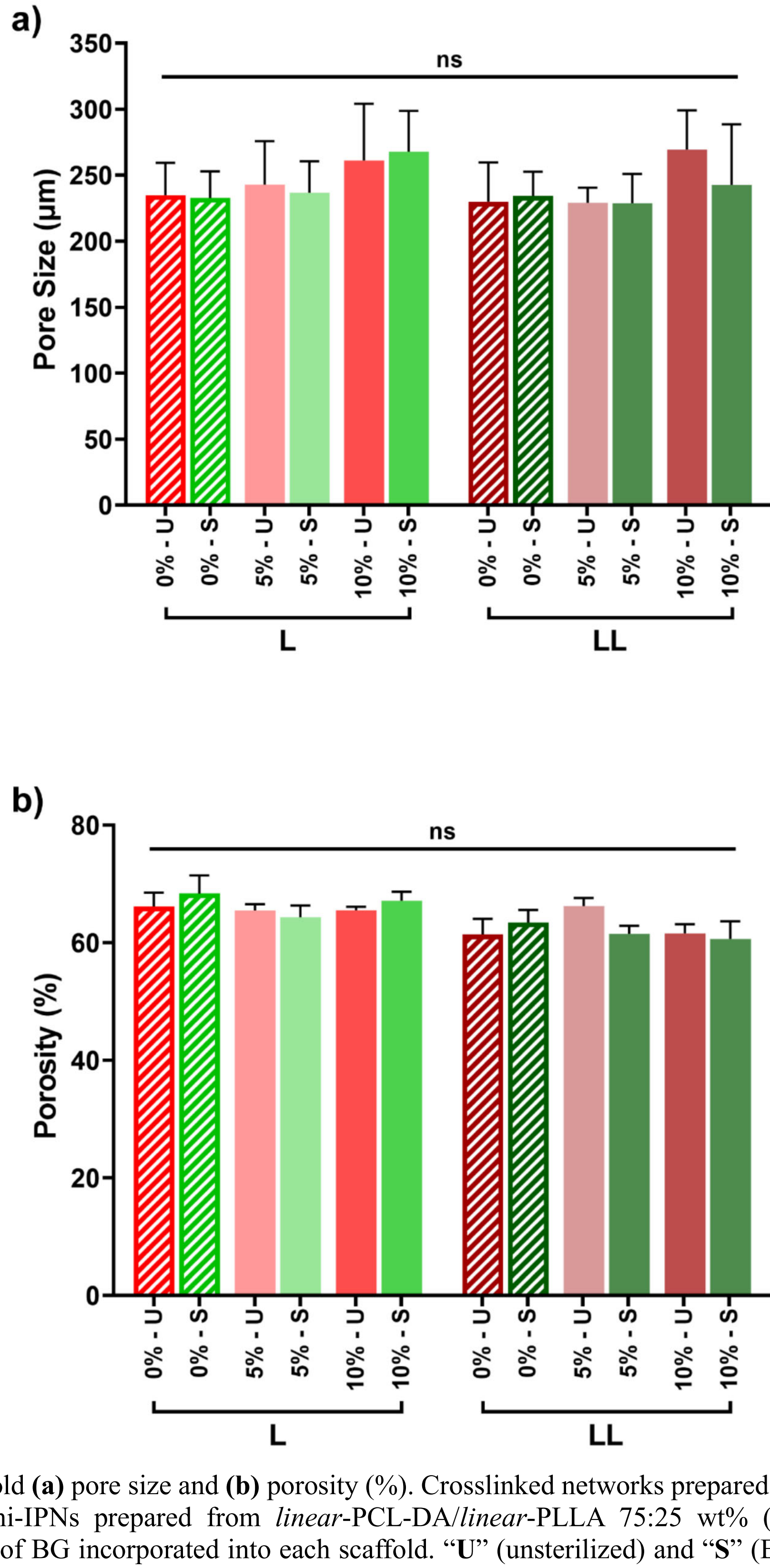

Figure S3. Scaffold **(a)** pore size and **(b)** porosity (%). Crosslinked networks prepared from *linear*-PCL-DA (**L**) and semi-IPNs prepared from *linear*-PCL-DA/*linear*-PLLA 75:25 wt% (**LL**). Percentages indicate the wt% of BG incorporated into each scaffold. "**U**" (unsterilized) and "**S**" (E-beam sterilized). *ns* = no statistical difference vs. analogous unsterilized scaffold.

Table S2. Scaffold pore size and porosity (%). Crosslinked networks prepared from *linear*-PCLDA (**L**) and semi-IPNs prepared from *linear*-PCL-DA/*linear*-PLLA 75:25 wt% (**LL**). Percentages indicate the wt% of BG incorporated into each scaffold. "**U**" (unsterilized) and "**S**" (E-beam sterilized).

Scaffold	Pore Size (μm)	Porosity (%)
**L-0%-U**	234.90 ± 24.41	66.17 ± 2.38
**L-0%-S**	232.70 ± 20.25	68.36 ± 3.10
**L-5%-U**	242.70 ± 33.13	65.48 ± 1.09
**L-5%-S**	236.80 ± 23.75	64.32 ± 2.01
**L-10%-U**	261.20 ± 42.92	65.50 ± 0.63
**L-10%-S**	267.90 ± 30.73	67.14 ± 1.52
**LL-0%-U**	229.80 ± 29.85	61.42 ± 2.64
**LL-0%-S**	234.30 ± 18.37	63.47 ± 2.13
**LL-5%-U**	229.00 ± 11.59	66.22 ± 1.42
**LL-5%-S**	228.70 ± 22.19	61.54 ± 1.34
**LL-10%-U**	269.50 ± 29.68	61.59 ± 1.55
**LL-10%-S**	242.70 ± 45.82	60.66 ± 2.99

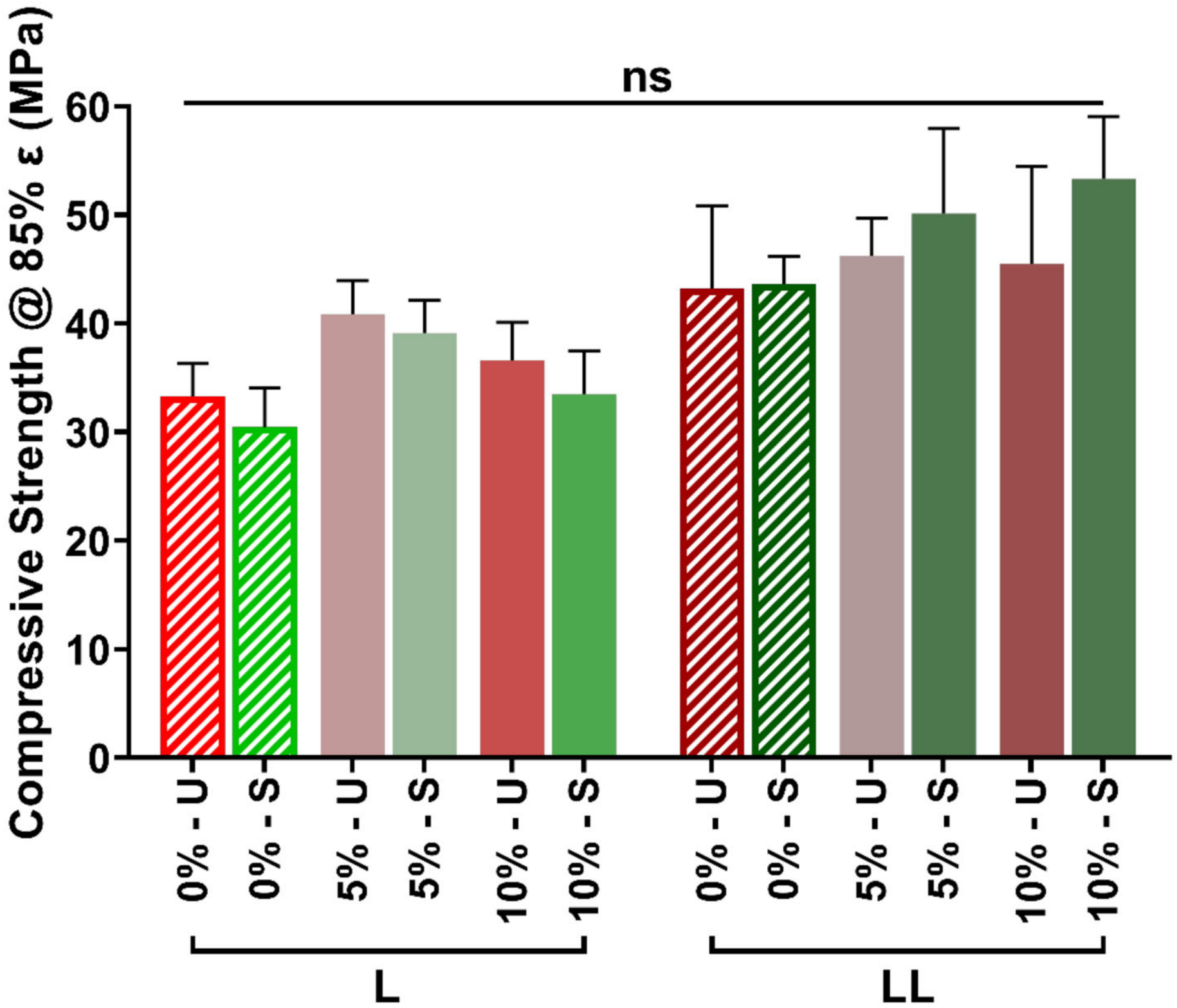

Figure S4. Compressive strength (*CS*) of scaffolds: Crosslinked networks prepared from *linear*-PCLDA (**L**) and semi-IPNs prepared from *linear*-PCL-DA/*linear*-PLLA 75:25 wt% (**LL**). Percentages indicate the wt% of BG incorporated into each scaffold. "**U**" (unsterilized) and "**S**" (E-beam sterilized). *ns* = no statistical difference vs. analogous unsterilized scaffold.

Table S3. Compressive strength (*CS*) of scaffolds: Crosslinked networks prepared from *linear*-PCLDA (**L**) and semi-IPNs prepared from *linear*-PCL-DA/*linear*-PLLA 75:25 wt% (**LL**). Percentages indicate the wt% of BG incorporated into each scaffold. "**U**" (unsterilized) and "**S**" (E-beam sterilized).

Scaffold	Modulus (MPa)	Strength (MPa)	Toughness (MJ m^{-3})
**L-0%-U**	5.13 ± 1.45	33.30 ± 3.00	4.98 ± 0.51
**L-0%-S**	5.38 ± 1.40	30.44 ± 3.61	4.44 ± 0.50
**L-5%-U**	8.28 ± 1.62	40.88 ± 3.07	5.96 ± 0.55
**L-5%-S**	7.17 ± 1.29	39.12 ± 2.99	5.92 ± 0.43
**L-10%-U**	5.36 ± 2.23	36.59 ± 3.53	5.49 ± 0.82
**L-10%-S**	4.24 ± 1.30	33.48 ± 3.98	4.53 ± 0.97
**LL-0%-U**	9.82 ± 4.93	43.22 ± 7.59	7.84 ± 1.83
**LL-0%-S**	8.92 ± 2.74	43.63 ± 2.55	7.15 ± 0.29
**LL-5%-U**	12.17 ± 3.58	46.25 ± 3.48	7.84 ± 0.86
**LL-5%-S**	12.68 ± 3.73	50.14 ± 7.82	8.34 ± 1.16
**LL-10%-U**	8.33 ± 3.46	45.47 ± 9.03	7.24 ± 1.40
**LL-10%-S**	7.06 ± 1.38	53.33 ± 5.73	7.29 ± 0.98

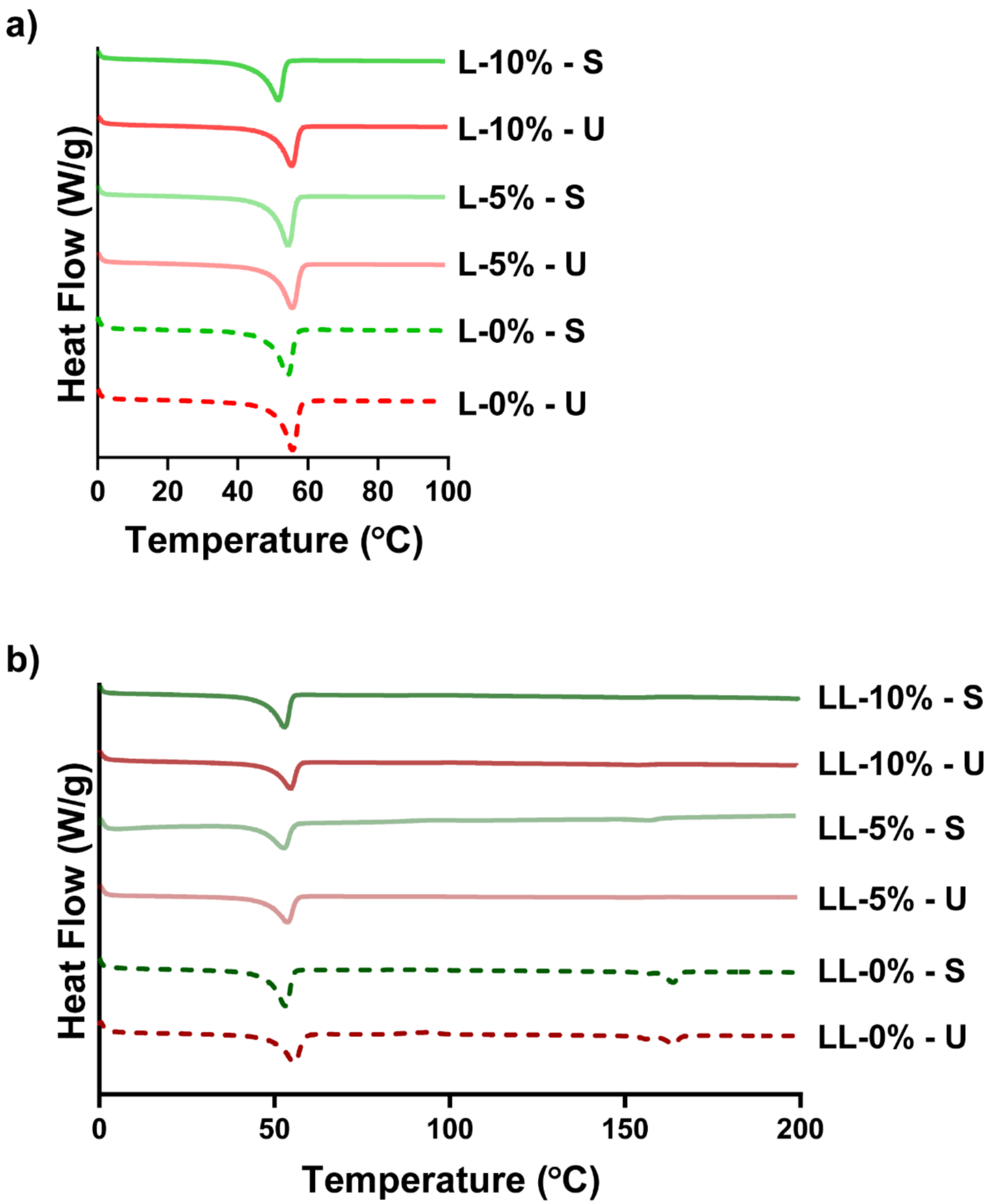

Figure S5. DSC thermograms of scaffolds: **(a)** Crosslinked networks prepared from *linear*-PCLDA (**L**) and **(b)** semi-IPNs prepared from *linear*-PCL-DA/*linear*-PLLA 75:25 wt% (**LL**). Percentages indicate the wt% of BG incorporated into each scaffold. "**U**" (unsterilized) and "**S**" (E-beam sterilized).

Table S4. Scaffold PCL and PLLA T_m and % crystallinity data: Crosslinked networks prepared from *linear*-PCL-DA (**L**) and semi-IPNs prepared from *linear*-PCL-DA/*linear*-PLLA 75:25 wt% (**LL**). Percentages indicate the wt% of BG incorporated into each scaffold. "**U**" (unsterilized) and "**S**" (E-beam sterilized).

**Scaffold**	**PCL**		**PLLA**	
	T_m **(°C)**	**%** X_c	T_m **(°C)**	**%** X_c
**L-0%-U**	55.31 ± 0.28	35.01 ± 0.55	-	-
**L-0%-S**	54.32 ± 0.22	34.49 ± 0.70	-	-
**L-5%-U**	55.81 ± 0.29	33.19 ± 0.94	-	-
**L-5%-S**	54.26 ± 0.07	36.26 ± 0.72	-	-
**L-10%-U**	55.74 ± 1.16	30.76 ± 2.08	-	-
**L-10%-S**	51.78 ± 0.51	27.89 1.41	-	-
**LL-0%-U**	55.50 ± 0.31	30.27 ± 1.10	163.83 ± 0.21	34.48 ± 1.08
**LL-0%-S**	53.24 ± 0.11	35.11 ± 0.98	163.43 ± 0.37	38.27 ± 2.67
**LL-5%-U**	54.06 ± 0.31	27.90 ± 0.79	-	-
**LL-5%-S**	52.77 ± 0.67	27.14 ± 1.00	-	-
**LL-10%-U**	54.47 ± 0.40	28.01 ± 1.12	-	-
**LL-10%-S**	52.60 ± 0.31	31.14 ± 1.40	-	-

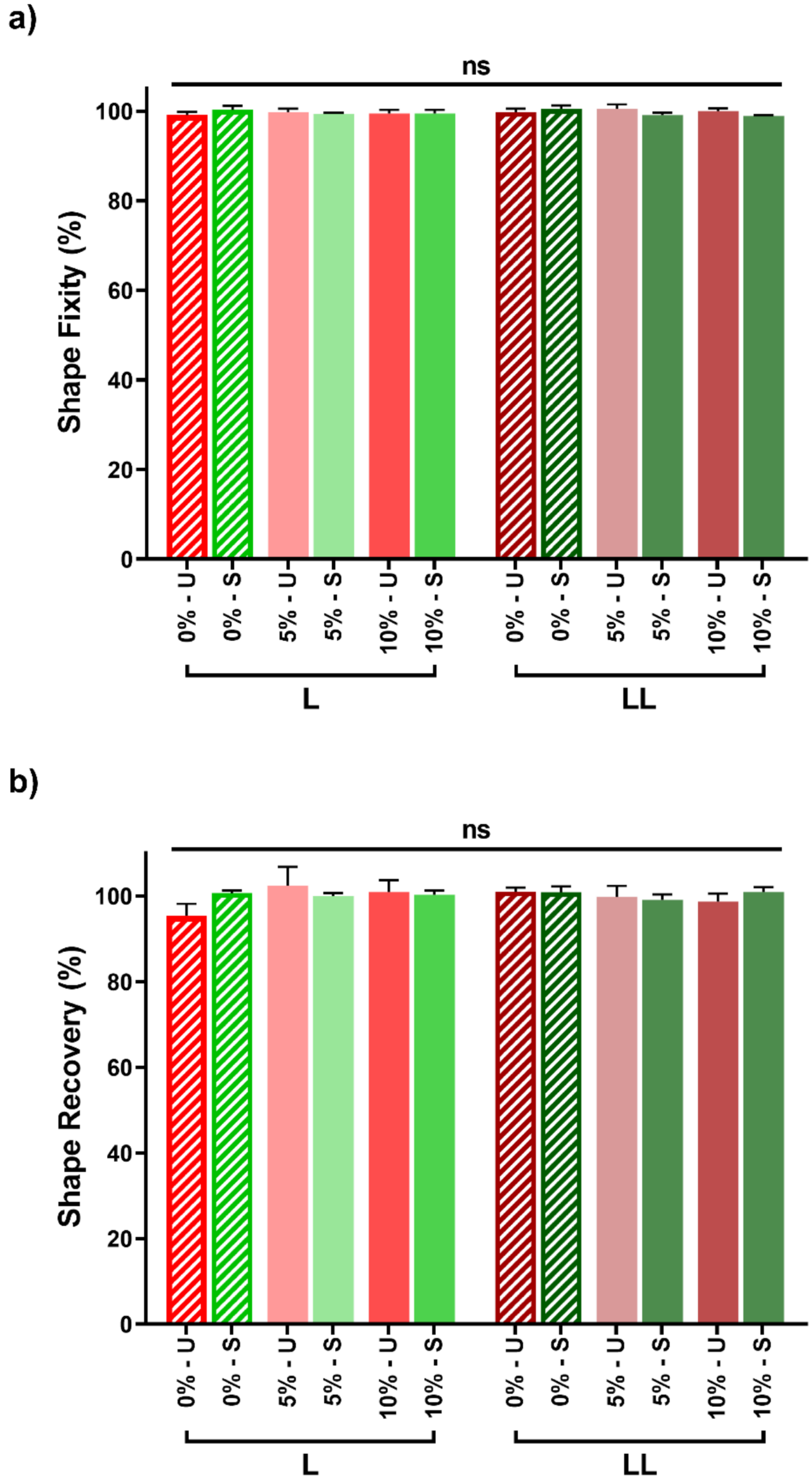

Figure S6. Scaffold **(a)** R_f (%) and **(b)** R_r (%): Crosslinked networks prepared from *linear*-PCL-DA (**L**) and semi-IPNs prepared from *linear*-PCL-DA/*linear*-PLLA 75:25 wt% (**LL**). Percentages indicate the wt% of BG incorporated into each scaffold. "**U**" (unsterilized) and "**S**" (E-beam sterilized). *ns* = no statistical difference vs. analogous unsterilized scaffold.

Table S5. Scaffold **(a)** R_f (%) and **(b)** R_r (%): Crosslinked networks prepared from *linear*-PCL-DA (**L**) and semi-IPNs prepared from *linear*-PCL-DA/*linear*-PLLA 75:25 wt% (**LL**). Percentages indicate the wt% of BG incorporated into each scaffold. "**U**" (unsterilized) and "**S**" (E-beam sterilized).

Scaffold	Shape Fixity (R_f) (%)	Shape Recovery (R_r) (%)
**L-0%-U**	99.12 ± 0.71	95.41 ± 2.76
**L-0%-S**	100.27 ± 0.92	100.65 ± 0.71
**L-5%-U**	99.80 ± 0.73	102.44 ± 4.35
**L-5%-S**	99.39 ± 0.20	100.06 ± 0.64
**L-10%-U**	99.46 ± 0.85	100.96 ± 2.73
**L-10%-S**	99.46 ± 0.85	100.28 ± 0.99
**LL-0%-U**	99.80 ± 0.73	101.02 ± 0.94
**LL-0%-S**	100.47 ± 0.77	100.91 ± 1.32
**LL-5%-U**	100.54 ± 0.92	99.87 ± 2.53
**LL-5%-S**	99.12 ± 0.51	99.10 ± 1.30
**LL-10%-U**	100.00 ± 0.61	98.69 ± 1.89
**LL-10%-S**	98.92 ± 0.23	100.95 ± 1.10

Table S6. Scaffold mass remaining (%) over time (0.1 M NaOH, 37 °C): Crosslinked networks prepared from linear-PCL-DA (**L**) and semi-IPNs prepared from linear-PCL-DA/linear-PLLA 75:25 wt% (**LL**). Percentages indicate the wt% of BG incorporated into each scaffold. "**U**" (unsterilized) and "**S**" (E-beam sterilized). (Note: -- indicates no mass remaining)

Scaffold	3 days	6 days	8 days	10 days	12 days	14 days	17 days
**L-0%-U**	96.09 ± 1.48	95.15 ± 1.51	94.44 ± 1.63	88.00 ± 0.73	84.44 ± 0.85	81.64 ± 2.45	51.12 ± 13.62
**L-0%-S**	96.57 ± 2.35	95.09 ± 2.11	91.49 ± 1.54	84.18 ± 0.89	79.34 ± 4.02	75.33 ± 5.33	34.87 ± 6.37
**L-5%-U**	90.12 ± 1.91	88.08 ± 1.90	76.89 ± 13.87	74.00 ± 2.38	66.17 ± 2.65	63.29 ± 8.00	28.43 ± 10.61
**L-5%-S**	94.18 ± 1.11	87.55 ± 2.61	82.64 ± 3.85	67.81 ± 2.95	63.73 ± 5.31	65.75 ± 7.31	23.49 ± 5.43
**L-10%-U**	91.80 ± 1.18	86.45 ± 0.65	81.94 ± 0.18	74.96 ± 0.99	67.81 ± 5.10	64.88 ± 2.74	40.35 ± 7.35
**L-10%-S**	87.46 ± 3.42	72.83 ± 2.12	64.39 ± 5.00	48.86 ± 3.79	33.36 ± 10.55	15.18 ± 9.03	0.00 ± 0.00
**LL-0%-U**	66.40 ± 5.96	27.89 ± 18.69	39.03 ± 6.69	36.33 ± 7.40	21.21 ± 19.46	11.68 ± 6.48	--
**LL-0%-S**	69.01 ± 9.55	57.65 ± 1.10	50.55 ± 7.15	27.93 ± 6.45	18.26 ± 12.33	11.39 ± 19.73	--
**LL-5%-U**	69.10 ± 1.75	61.26 ± 14.55	26.00 ± 5.97	19.82 ± 4.99	19.69 ± 9.25	--	--
**LL-5%-S**	66.17 ± 4.11	34.73 ± 14.62	34.79 ± 10.24	22.34 ± 10.71	18.72 ± 6.69	--	--
**LL-10%-U**	68.97 ± 2.37	49.84 ± 9.65	39.98 ± 4.33	26.18 ± 7.32	16.80 ± 13.80	--	--
**LL-10%-S**	64.66 ± 2.01	41.49 ± 9.74	29.82 ± 2.70	12.14 ± 5.69	5.73 ± 5.30	0.84 ± 1.45	--

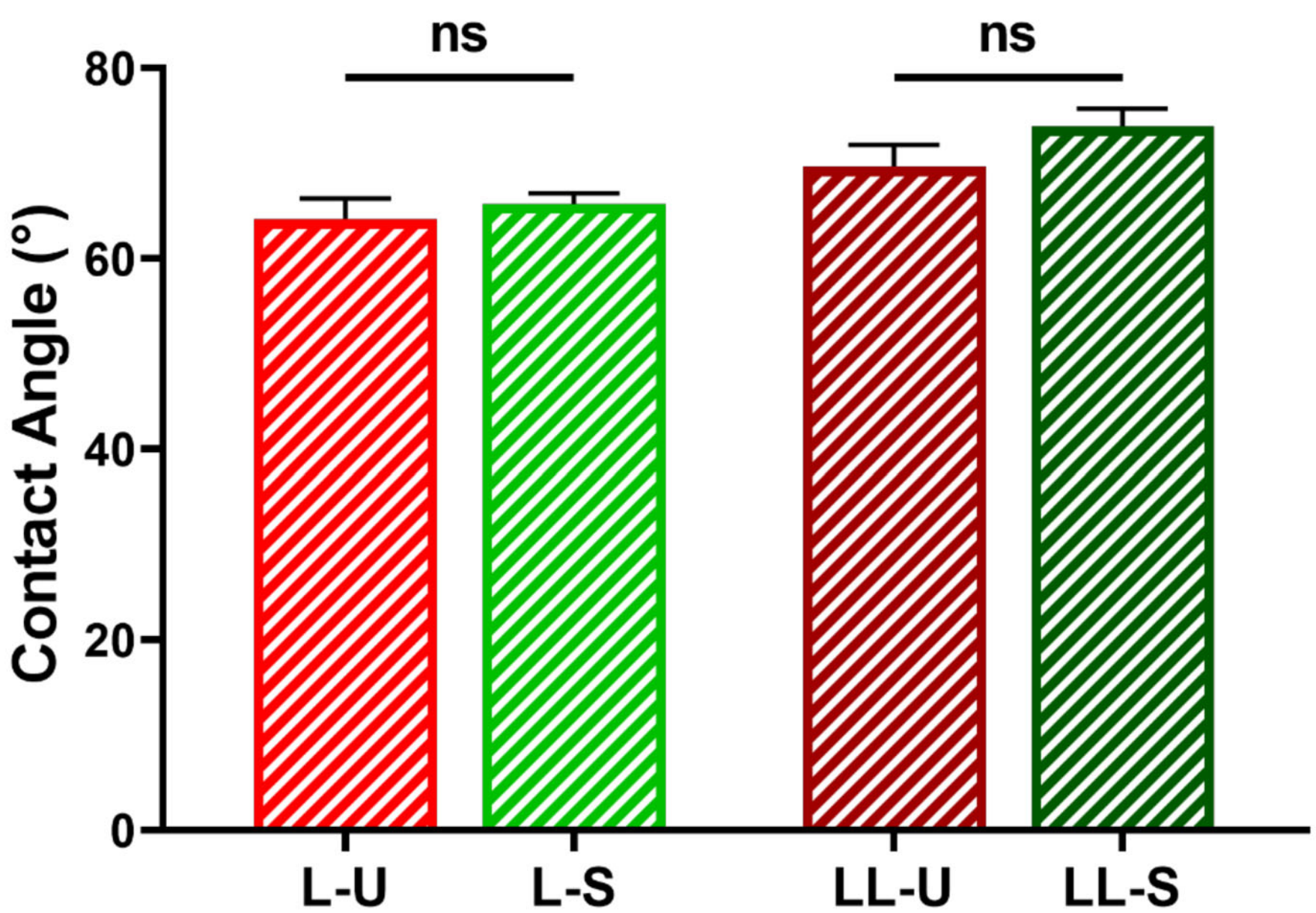

Figure S7. Contact angles of films: Crosslinked networks prepared from *linear*-PCL-DA (**L**) and semi-IPNs prepared from *linear*-PCL-DA/*linear*-PLLA 75:25 wt% (**LL**). Percentages indicate the wt% of BG incorporated into each scaffold. "**U**" (unsterilized) and "**S**" (E-beam sterilized). *ns* = no statistical difference vs. analogous unsterilized film.

Table S7. Contact angles of films: Crosslinked networks prepared from *linear*-PCL-DA (**L**) and semi-IPNs prepared from *linear*-PCL-DA/*linear*-PLLA 75:25 wt% (**LL**). Percentages indicate the wt% of BG incorporated into each scaffold. "**U**" (unsterilized) and "**S**" (E-beam sterilized).

Film	Contact Angle (°)
**L-0%-U**	64.14 ± 2.17
**L-0%-S**	65.71 ± 1.12
**LL-0%-U**	69.63 ± 2.33
**LL-0%-S**	73.90 ± 1.86

Table S8. Surface roughness (*Sa*) values for films: Crosslinked networks prepared from *linear*-PCL-DA (**L**) and semi-IPNs prepared from *linear*-PCL-DA/*linear*-PLLA 75:25 wt% (**LL**). Percentages indicate the wt% of BG incorporated into each scaffold. "**U**" (unsterilized) and "**S**" (E-beam sterilized).

Film	***Sa* (µm)**
**L-0%-U**	0.10 ± 0.03
**L-0%-S**	0.07 ± 0.03
**LL-0%-U**	0.25 ± 0.10
**LL-0%-S**	0.27 ± 0.07

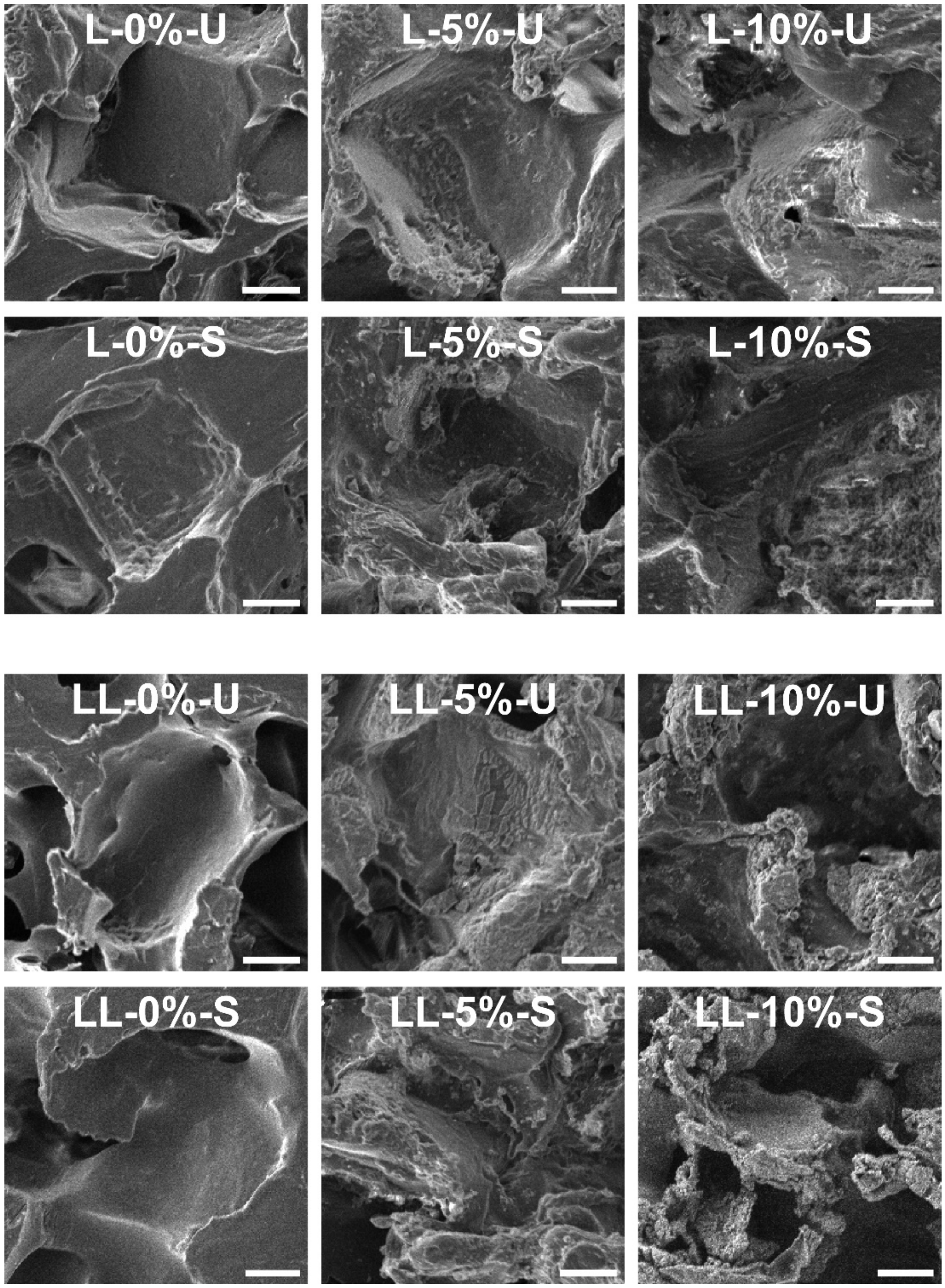

Figure S8. SEM images of scaffolds following 1 day in SBF (1X): Crosslinked networks prepared from *linear*-PCL-DA (**L**) and semi-IPNs prepared from *linear*-PCL-DA/*linear*-PLLA 75:25 wt% (**LL**). Percentages indicate the wt% of BG incorporated into each scaffold. "**U**" (unsterilized) and "**S**" (E-beam sterilized). (Scale bars = 50 μm).

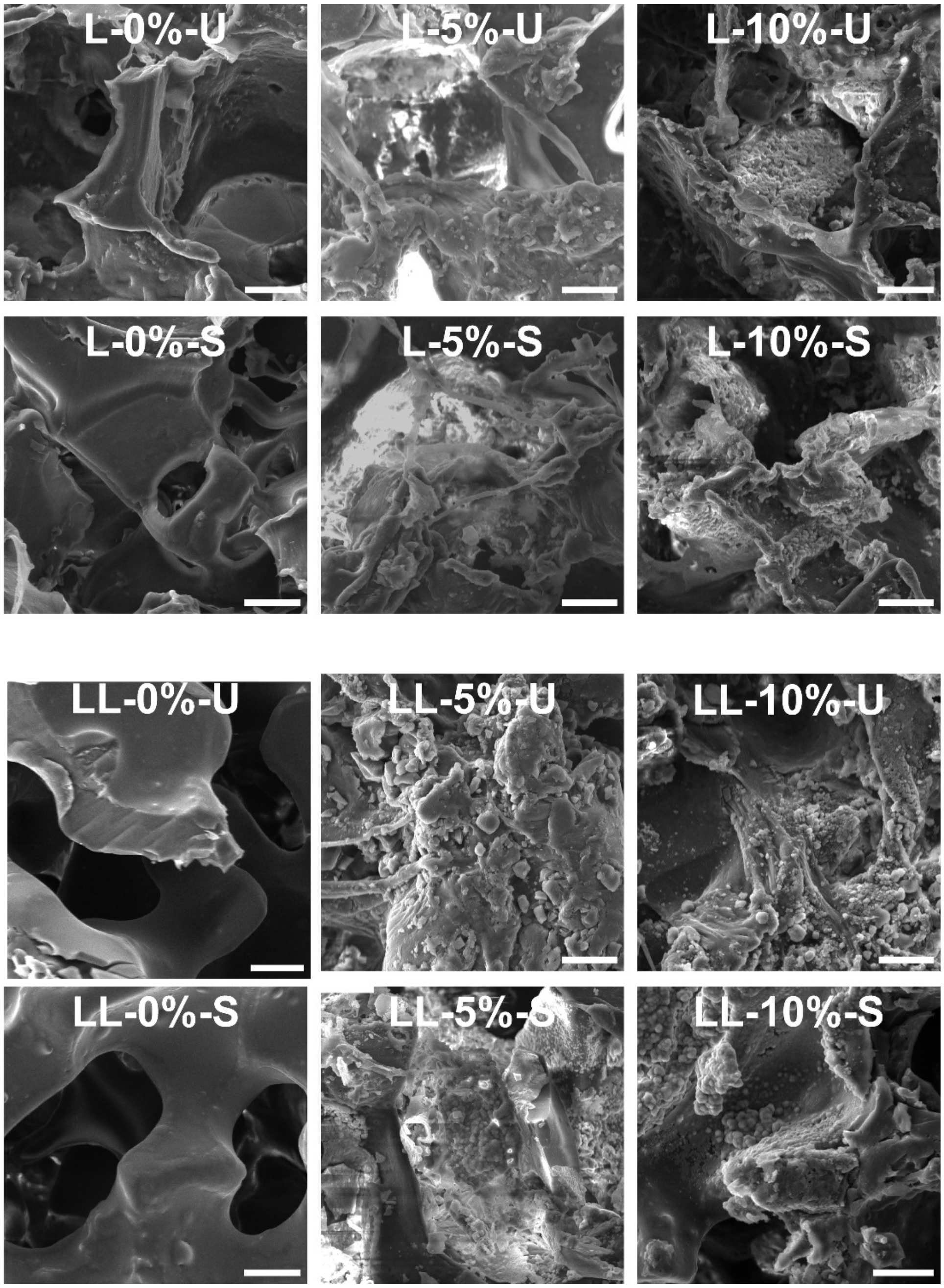

Figure S9. SEM images of scaffolds following 2 weeks in SBF (1X): Crosslinked networks prepared from *linear*-PCL-DA (**L**) and semi-IPNs prepared from *linear*-PCL-DA/*linear*-PLLA 75:25 wt% (**LL**). Percentages indicate the wt% of BG incorporated into each scaffold. "**U**" (unsterilized) and "**S**" (E-beam sterilized). (Scale bars = 50 μm).

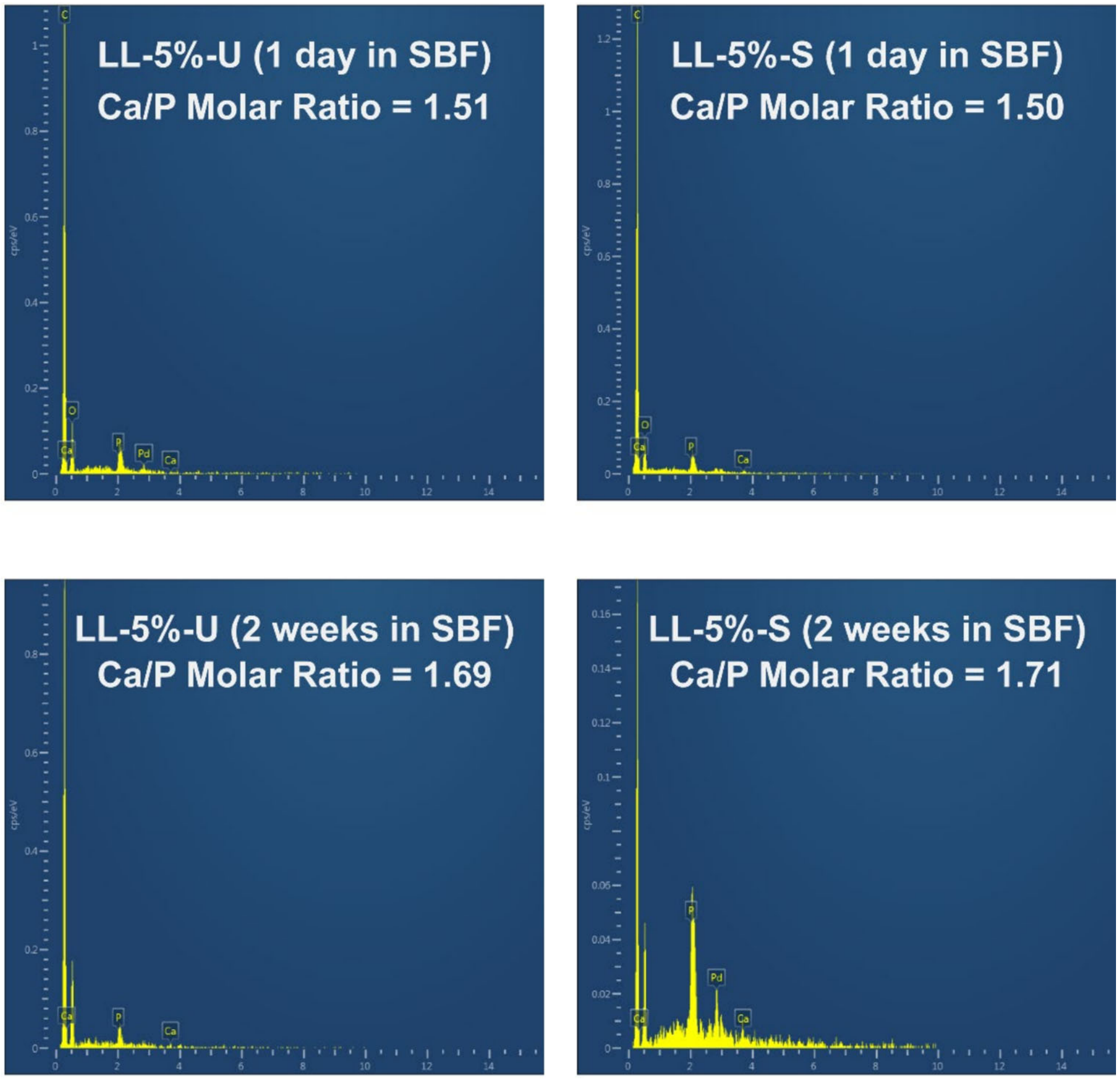

Figure S10. Representative EDS spectra of **LL-5%** scaffolds [unsterilized (**U**) and sterilized (**S**)], after 1 day and 2 weeks of SBF (1X) exposure.
